# Pan-genome analysis of the glutathione S-transferase gene family reveals the core and dispensable components of GST evolution in *Arachis*

**DOI:** 10.3389/fpls.2026.1986683

**Published:** 2026-10-26

**Authors:** Congbo Huang, Yusong Zhang, Yuan Jiang, Yanqing Luo, Kaiqin Zhao, Genze Li, Xiaoyan Yuan, Minglian Fu

**Affiliations:** 1 Industrial Crops Research Institute, Yunnan Academy of Agricultural Sciences, Kunming, China; 2 Yunnan Key Laboratory of Genetic Improvement of Herbal Oil Crops, Industrial Crops Research Institute, Yunnan Academy of Agricultural Sciences, Kunming, China; 3 College of Agriculture, Yunnan University, Kunming, China

**Keywords:** *Arachis*, core and dispensable genes, gene family evolution, glutathione S-transferase (GST), orthologous gene groups, pan-genome, peanut

## Abstract

**Introduction:**

Gene-family identification and comparative analyses have largely relied on a single reference genome and therefore cannot capture presence/absence variation among genomes.

**Methods:**

The glutathione S-transferase (GST) family was analysed computationally across 20 *Arachis* genomes, 16 allotetraploid and four from the diploid progenitor lineages, using orthologous gene groups (OGGs) as the unit of analysis. Gene structure, phylogeny, duplication, collinearity, Ka/Ks and expression in tissue and stress transcriptomes were compared among OGG categories.

**Results:**

The 1,436 GSTs carrying both canonical domains were assigned to 57 OGGs, comprising 18 core OGGs present in all 20 genomes and 39 dispensable OGGs (softcore, shell and line-specific). The family was structurally uniform, and its uniformity declined from the core to the shell OGGs. The OGGs fell into the Tau, Phi, Theta and Lambda subfamilies, 53 of the 57 belonging to Tau. WGD/segmental duplicates predominated in core and softcore OGGs, making up 65.2% of core genes, whereas tandem duplicates predominated among shell genes at 42.0%, and WGD/segmental duplicates made up a far larger share of the GSTs in the tetraploids than in the diploids. Core and softcore OGGs occupied syntenic positions shared with the progenitor diploids, about half of the cross-genome syntenic links connecting the A and B subgenomes. Among 23,399 within-OGG gene pairs, mean Ka/Ks was below 1 in 43 of the 46 OGGs for which it could be estimated, consistent with predominantly purifying selection; no pair with Ka/Ks above 1 departed significantly from neutrality, and neither Ka, Ks nor Ka/Ks differed between core and dispensable OGGs. Across 22 tissues of the cultivar NDH108, all 35 core genes were expressed, the nine silent genes belonged to softcore or shell OGGs, and the calyx was the most frequent tissue of peak expression. Under drought, cold or salt, 74 of the 84 NDH108 GSTs responded, at similar rates in core and dispensable OGGs.

**Discussion:**

All results are computational and were not validated experimentally. The OGG-based analysis resolves variation in GST content and genomic organisation across the *Arachis* panel that a single reference genome does not capture, and the genes it highlights are candidates for experimental study.

## 1 Introduction

Glutathione S-transferases (GSTs) constitute a ubiquitous, multigene superfamily of enzymes that conjugate the tripeptide glutathione (GSH) to electrophilic centres of a broad range of substrates. A canonical GST comprises two domains: an N-terminal thioredoxin (TRX)-like domain (Pfam PF02798, GST_N) and a C-terminal all-α-helical domain (Pfam PF00043, GST_C) (Sylvestre-Gonon et al., 2019). On the basis of this domain organisation and phylogeny, plant GSTs have been classified into as many as 14 classes, which can be further distinguished by whether the catalytic residue is the ancestral cysteine (Cys-GSTs) or a serine (Ser-GSTs); the plant-specific Tau (U) and Phi (F) classes are by far the most expanded, and plant GSTs frequently occur in tandem gene clusters, a feature associated with functional diversification among closely related members (Sylvestre-Gonon et al., 2019; Ugalde et al., 2025).

Plant GSTs are a family of functionally diverse detoxification enzymes (Oakley, 2011) that participate in the detoxification of herbicides and xenobiotics (Dixon et al., 2010; Lin et al., 2025). Beyond their detoxification role, GSTs can act as glutathione peroxidases (GPOX) to reduce lipid hydroperoxides, thereby regulating reactive oxygen species (ROS) levels and alleviating oxidative stress (Ugalde et al., 2025). Cysteine-containing GSTs (such as DHAR) can reduce dehydroascorbate to ascorbate, contributing to cellular redox homeostasis (Lallement et al., 2014). GST proteins are also involved in the binding, transport, and metabolism of flavonoids (such as anthocyanins), thereby linking this family to plant secondary metabolism (Dixon et al., 2010; Manzoor et al., 2023; Zhao, 2015); in *Arabidopsis*, AtGSTF12 (TT19) is required for the deposition of anthocyanins (Dixon et al., 2010).In peanut, GSTs are associated with a range of stress responses. Root GST activity increases markedly under arsenate stress and has been proposed as a biomarker of metalloid toxicity (Bianucci et al., 2017). *AhGST23*, a gene positioned within the ascorbate–glutathione (AsA–GSH) cycle, was recently shown to mediate drought and salt stress tolerance; silencing this gene disrupts glutathione-related metabolism and impairs ROS scavenging capacity (Li et al., 2026).

Reflecting this importance, the GST family has been characterised genome-wide in several species, with markedly different family sizes reported. For example, 53 GST members have been identified in *Arabidopsis thaliana* (Sappl et al., 2004), 79 in rice (Jain et al., 2010), 330 in wheat (Wang et al., 2019a), 126 in soybean (Hasan et al., 2020), 90 in potato (Islam et al., 2018), 85 in pepper (Islam et al., 2019) and 120 in quinoa (Tiwari et al., 2023). In peanut, a single-reference survey identified 163 *GST* genes assigned to six classes (Wang et al., 2019b). However, all of these analyses describe the family within one reference genome and therefore treat it as a fixed gene set, which cannot capture the gene presence/absence variation (PAV) and copy-number variation (CNV) that exist among accessions of a species.

Cultivated peanut (*Arachis hypogaea* L.) is a globally important oil and protein crop, with a high kernel oil content (45–56%) and high-quality protein (22–30%); as a nitrogen-fixing legume it also improves soil fertility. China is the world’s largest producer and consumer of peanut, accounting for 38.8% of global production and 51.9% of peanut oil output in 2024 (Zhao et al., 2026). Peanut is an allotetraploid (AABB, 2n = 4x = 40) with a large (~2.54–2.62 Gb), repeat-rich genome that arose from hybridisation between the A-genome diploid *A. duranensis* and the B-genome diploid *A. ipaensis* followed by chromosome doubling (Bertioli et al., 2016, 2019; Zhao et al., 2026); multiple lines of evidence indicate that this polyploidisation occurred recently, within the past few thousand years, rather than at a more ancient date (Bertioli et al., 2020; Bian et al., 2026).Over the past decade, peanut genome resources have accumulated rapidly, providing a basis for analyses that move beyond a single reference genome (Zhao et al., 2026). In 2016, the genomes of the two diploid progenitors *A. duranensis* and *A. ipaensis* were the first to be released (Bertioli et al., 2016). Chromosome-scale reference genomes of cultivated tetraploids were subsequently completed, including Tifrunner (Bertioli et al., 2019), Shitouqi (Zhuang et al., 2019) and Fuhuasheng (Chen et al., 2019), and a high-quality genome of the wild allotetraploid *A. monticola* was also assembled (Yin et al., 2018). More recently, telomere-to-telomere (T2T) assemblies have further improved genome completeness, as exemplified by the T2T genome of the cultivar Yuanza9102 (Wang et al., 2025) and by T2T assemblies of two diploid and four tetraploid varieties combined with resequencing of 521 accessions (Bian et al., 2026). In parallel, peanut pan-genomes built from multiple high-quality genomes and hundreds of resequenced accessions have revealed genome-wide structural variation associated with agronomic traits (Zhao et al., 2025), and graph-based pan-genome analysis has resolved loci that are difficult to detect from a single reference (Thompson et al., 2025). In addition, the PeanutOmics platform, integrating multi-omics data across 22 tissues, provides a resource for gene-expression analysis (Xue et al., 2026). Together, these chromosome-scale *Arachis* genomes make it feasible to characterise a gene family systematically at the pan-genome scale.

The pan-genome concept partitions genes into a core component shared by all individuals and a dispensable component present in only some individuals (Tettelin et al., 2005). Although this framework was originally proposed at the whole-genome level, it has recently been applied at the level of a single gene family, using orthologous gene groups (OGGs) as the unit of classification. For example, across 20 barley genomes, bHLH genes were assigned to 201 OGGs and further classified as core, softcore, shell and lineage-specific (Tong et al., 2025); comparable analyses have been reported for the WRKY family across 22 tea (*Camellia sinensis*) genomes (Wang et al., 2026) and for the auxin response factor (ARF) family in barley (Tan et al., 2026). Applying this framework to a stress-related family such as the *GST*s across multiple peanut genomes makes it possible to distinguish the conserved component of the family from the variable one, and also helps to mitigate the annotation biases inherent in single-genome analyses.

In this study we used the whole genome protein sets of 20 *Arachis* genomes, spanning the AABB, AA and BB genome types, to identify and analyse the *GST* family systematically within a pan-genome framework. We first identified *GST* members in each genome and constructed an *Arachis* GST pan-gene family comprising 1,436 genes in 57 orthologous gene groups, and then partitioned these orthologous gene groups into core and dispensable components. Building on this classification, we examined the subfamily assignment, copy-number variation and conserved motif architecture of each component, inferred the modes of gene duplication together with the selective pressure acting on duplicated gene pairs (Ka/Ks), and analysed the tissue expression of the GSTs of the cultivar NDH108 and their responses in three public stress transcriptomes. All analyses were computational. This study aims to characterise the peanut GST family at the pan-genome level, to distinguish its conserved core component from its variable dispensable component, and to resolve the duplication and selection processes that have shaped this composition; the results provide a pan-genome level resource for understanding the evolution of this family and a basis for selecting candidate genes for subsequent functional studies.

## 2 Materials and methods

### 2.1 Plant genome data sources

A total of 20 *Arachis* genomes were collected, comprising 16 allotetraploid cultivated or wild accessions (AABB, 2n=4x=40), 3 A-genome diploids (AA) and 1 B-genome diploid (BB). The four diploids belong to the A- and B-genome progenitor lineages of cultivated peanut and are not accessions of *A. hypogaea*. The protein sequences, coding sequences (CDS) and genome annotation files of these genomes were downloaded from NCBI (https://www.ncbi.nlm.nih.gov/datasets/genome/?taxon=3817), the peanut pan-genome database PeanutPan (https://cgm.sjtu.edu.cn/PeanutPan/index.html), GigaDB (https://gigadb.org/dataset/100453) and figshare (https://doi.org/10.6084/m9.figshare.26551309.v1). The name, genome type and source of each genome are listed in **Supplementary Table S1**.

### 2.2 Identification of the *GST* gene family

For each genome, the protein sequence corresponding to the longest transcript of every gene was first extracted with TBtools (Chen et al., 2020). A GST protein was required to contain both the N-terminal domain (GST_N, Pfam PF02798) and the C-terminal domain (GST_C, Pfam PF00043) of GST. The hidden Markov models of these two domains were downloaded from the Pfam database (https://www.ebi.ac.uk/interpro/entry/pfam) and searched against the protein set of each genome using hmmsearch in HMMER v3.4(E-value threshold 1e-5) (Eddy, 2011). Sequences hitting either model were pooled, giving 2,564 candidates, and submitted to the NCBI Batch CD-Search (E-value threshold 1e-5); the 1,529 sequences with specific hits to both an N-terminal and a C-terminal GST domain model were retained. A length window of 150 to 350 amino acids was then applied, removing 93 sequences, all longer than 350 amino acids and 71 of them elongation factor 1B gamma (EF1Bγ)-like proteins in which the GST-like domains are fused to further domains, and leaving 1,436 GSTs. Because both PF02798 and PF00043 are required, GST classes whose C-terminal domain is poorly matched by PF00043 can be missed by this procedure. The effect of this criterion on subfamily coverage was assessed by searching the 53 *Arabidopsis* GSTs with the two HMMs and by searching the Tifrunner proteome with BLASTP (BLAST+ v2.17.0) using the *Arabidopsis* GSTs as queries (E-value threshold 1e-10, five target sequences per query).

### 2.3 Analysis of gene structure

The structure of each *GST* was extracted from the GFF3 annotation of its genome of origin. Gene length was taken as the span of the mRNA feature, the number of CDS features per transcript gave the CDS number, the exon count minus one gave the intron number, and total intron length was taken as the mRNA span minus the summed exon length. Amino acid length was obtained from the protein sequence and independently recomputed from the summed CDS length; the two agreed to within one residue for all 1,436 genes. Metrics were summarised by genome and by OGG and their distributions compared with boxplots. Gene length and intron length are displayed up to the 98th percentile of the family distribution, because four genes lying across assembly gaps carry introns one to two orders of magnitude longer than the rest and would otherwise compress the remaining distributions; these genes are retained in all calculations and the groups containing them are marked in the figures.

### 2.4 Definition of pan-genome orthologous groups

Orthology was inferred with OrthoFinder v3.1.2 (Emms and Kelly, 2019) from the complete protein sets of the 20 genomes, one protein per gene and 1,312,427 proteins in total, with DIAMOND v2.1.24 (Buchfink et al., 2021)for the all-versus-all search, FAMSA v2.4.1 (Deorowicz et al., 2016) for multiple alignment and FastTree v2.2.0 (Price et al., 2010) for gene trees; the run produced 61,872 orthogroups. The 57 orthogroups containing at least one of the 1,436 GSTs were taken as the GST orthologous gene groups (OGGs). The occurrence of an OGG in a genome was scored from its GST members only; 23 of the 57 OGGs also contained proteins that had failed the GST criteria, 212 in total, and these were not counted. An OGG present in all 20 genomes was classified as core, one present in 18 or 19 genomes as softcore, one present in 3 to 17 genomes as shell and one present in no more than 2 genomes as line-specific, and the softcore, shell and line-specific OGGs were together termed dispensable. These categories describe the occurrence of an OGG across the 20-genome *Arachis* panel, which includes the diploid progenitor lineages, rather than across accessions of cultivated peanut, and they refer to the OGG rather than to its individual gene copies. The GSTs belonging to OGGs of each category are referred to as core, softcore, shell and line-specific genes.

### 2.5 Phylogenetic analysis and subfamily assignment

The species tree (**Figure 1**) was produced by the whole-proteome OrthoFinder run described above. OrthoFinder selected 488 orthogroups in which most genomes carried a single copy, concatenated their alignments into a matrix of 134,066 amino-acid columns, inferred the tree with FastTree and rooted it with STRIDE (Emms and Kelly, 2017); the node values are FastTree SH-like local supports. For the GST backbone tree, one member of each OGG was taken as its representative. The 57 representatives were aligned with the 53 GST proteins of *Arabidopsis thaliana* (https://www.arabidopsis.org/browse/gene_family/gst) using MAFFT v7.526 with the --auto option (Katoh and Standley, 2013). The alignment was trimmed with trimAl v1.5 (automated1) (Capella-Gutiérrez et al., 2009), leaving 159 columns, and a maximum-likelihood tree was constructed with IQ-TREE v3.1.1 (Wong et al., 2026) under the Q.YEAST+R4 model selected by ModelFinder according to BIC (Kalyaanamoorthy et al., 2017), with 1,000 ultrafast bootstrap replicates (Hoang et al., 2018). Based on the clustering of the representative and reference sequences in the phylogenetic tree, each OGG was assigned to a subfamily. As an independent check, every one of the 1,436 GSTs was searched with BLASTP against the 53 *Arabidopsis* GSTs and the subfamily of its best hit compared with that of its OGG. Conserved motifs (ten in total) were identified in the representative sequences with MEME v5.5.9 (Bailey et al., 2015) at https://meme-suite.org/meme/tools/meme with otherwise default settings.

**Figure 1:**
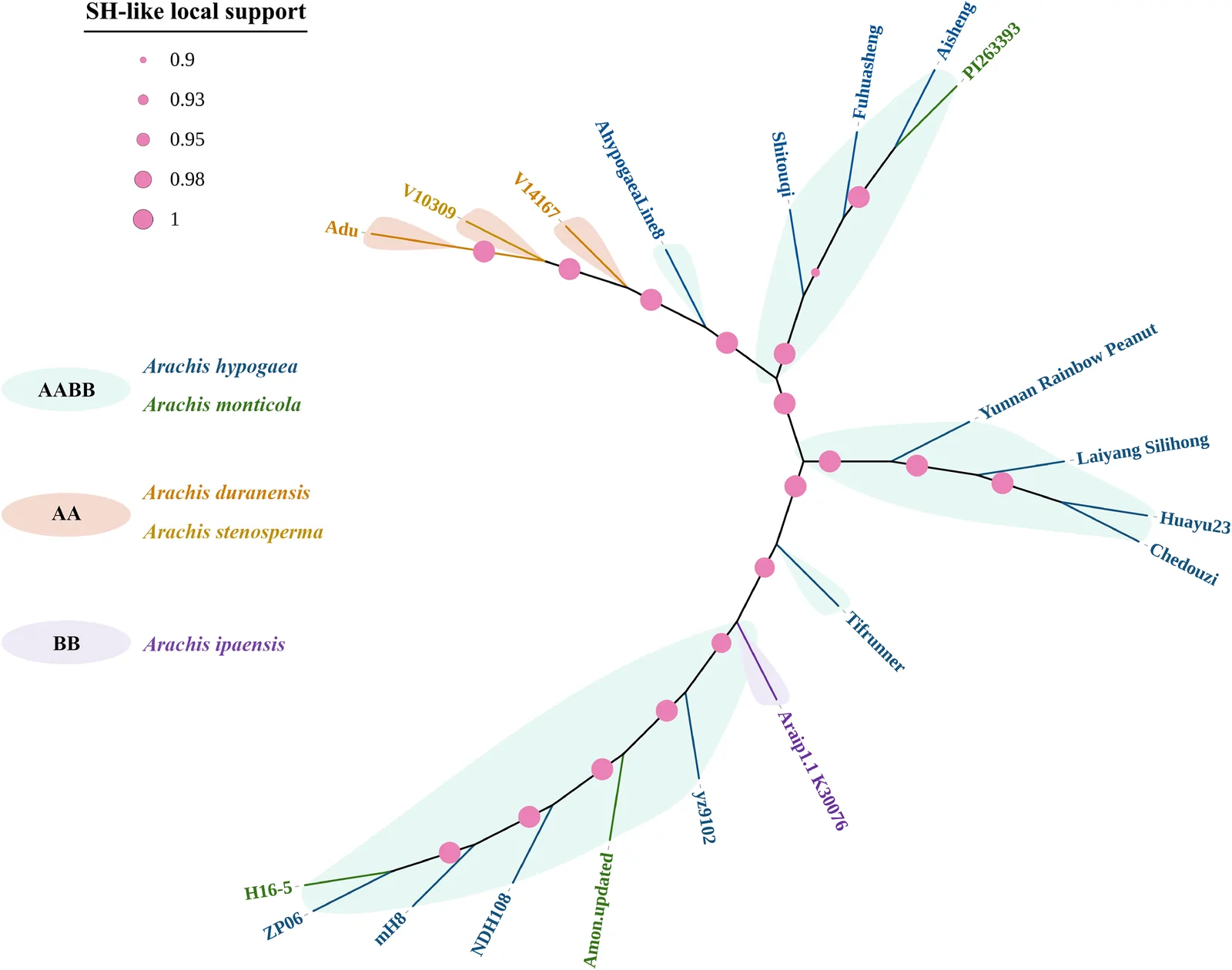
Species tree of the 20 *Arachis* genomes, inferred by OrthoFinder with FastTree from the concatenated alignment of 488 orthogroups of the complete proteomes (134,066 amino-acid columns) and rooted with STRIDE. Tip labels are colored by species, with *A. hypogaea* in blue and *A. monticola* in green (both AABB), *A. duranensis* in orange and *A. stenosperma* in olive (AA), and *A. ipaensis* in purple (BB). Background shading marks the AABB, AA and BB groups, and node dots are scaled by FastTree SH-like local support (0.90-1.00).

### 2.6 Gene duplication analysis

Duplication modes were assigned with MCScanX (Wang et al., 2012). Each of the 20 proteomes was searched against itself with BLASTP (BLAST+ v2.12.0) (Camacho et al., 2009) at an E-value threshold of 1e-5 with at most five target sequences per query, and the hits were supplied to MCScanX together with the gene coordinates taken from the GFF3 files. The duplicate_gene_classifier program places each gene in the highest-ranking mode for which it qualifies, ranked as WGD/segmental (anchor gene of a collinear block), tandem (a homologous gene immediately adjacent in gene order), proximal (a homologous gene fewer than 20 genes away), dispersed (any other homologous gene) and singleton (no homologous hit). The duplication type of every *GST* was then tabulated against its genome of origin and against its OGG, giving a genome-by-mode matrix and an OGG-by-mode matrix. Genomes were ordered by the species tree and OGGs by the backbone tree of the representative sequences, so that mode composition could be read against phylogenetic position, and counts were expressed both as absolute numbers and as percentages within each genome or OGG. The proximal mode is defined from the rank distance between genes along the annotation, so that differences in gene ordering between annotations affect how many pairs qualify; proximal and dispersed counts should therefore be compared between genomes with caution.

### 2.7 Collinearity analysis

Collinear relationships were examined at two scales. Within each of the 20 genomes, the collinear blocks reported by MCScanX from the all-versus-all alignment described above were screened for blocks whose anchors were both *GST*s, and the resulting pairs were drawn on circular plots of that genome with Matplotlib v3.11.0 (Hunter, 2007). Chromosome names were normalised across the six naming conventions used by the source annotations, and the 20 chromosomes of the tetraploids and 10 of the diploids carrying the most genes were assigned to A01-A10 and B01-B10. Between genomes, *A. duranensis*, *A. monticola*, *A. hypogaea* cv. Tifrunner and *A. ipaensis* were compared pairwise. Protein sequences were aligned between genomes with DIAMOND v2.2.5 and collinear blocks were called with MCScanX restricted to cross-genome blocks, and blocks whose anchors were both *GST*s assigned to the same OGG were retained. Pairs linking chromosomes of the same subgenome were distinguished from pairs linking the A and B subgenomes, which represent homoeologous rather than orthologous relationships, and the two sets were plotted separately. Collinear relationships within the Tifrunner genome were additionally visualized with the Advanced Circos module of TBtools (Chen et al., 2020).

### 2.8 Analysis of natural selection

For each OGG derived from the orthogroup analysis, all genes were compared pairwise, giving 23,399 within-OGG gene pairs. For each pair the two coding sequences were translated, the proteins aligned with MAFFT v7.526 (Katoh and Standley, 2013), and the original codons threaded back onto the protein alignment; codon columns containing a gap, an ambiguous base or a stop in either sequence were removed. Ka, Ks and Ka/Ks were then calculated from the resulting codon alignments with KaKs_Calculator v2.0 under the NG model (Nei and Gojobori, 1986; Wang et al., 2010). The NG model was chosen because most pairs were closely related, the median Ks of the non-identical pairs being 0.036 with a median of ten substitutions per pair, so that the additional parameters of more complex models could not be estimated reliably. Among the 23,399 pairs, 8,154 (34.9%) were identical in sequence, so that Ka and Ks were both zero and the ratio undefined, and a further 619 pairs (2.6%) carried only non-synonymous substitutions, giving Ks = 0 and an infinite ratio. Identical pairs contributed Ka = 0 and Ks = 0 to the Ka and Ks means, whereas pairs with an infinite ratio were excluded from the Ka/Ks means and counted separately. The Ka, Ks and Ka/Ks of an OGG were each taken as the arithmetic mean over its pairs, and the distribution of the pairwise values within each OGG was displayed as a ridgeline plot. Because the pairs within an OGG share genes and are not independent, OG0001327 alone contributing 2,145 pairs, the OGG mean rather than the individual pair was used as the unit of comparison. Pan-genome categories were collapsed into core and dispensable, and the two groups were compared with Welch’s t-test and the two-sided Mann–Whitney U test (Mann and Whitney, 1947). Departure from neutrality was tested for each pair with Fisher’s exact test on its synonymous and non-synonymous site and substitution counts, as reported by KaKs_Calculator, and the same test was computed for the pairs with Ks = 0 and Ka > 0, for which the program reports none.

### 2.9 Tissue expression analysis

Tissue expression was examined with the 22-tissue expression atlas of the cultivar NDH108 in PeanutOmics (https://cgm.sjtu.edu.cn/PeanutOmics/gene.html) (Xue et al., 2026). The analysis was carried out at the gene level for all 84 NDH108 GSTs, each carrying the OGG and pan-genome category of the OGG to which it belongs; 83 had expression records, NDH12G13621.1 having none. The FPKM values of the three replicates of each tissue were averaged, a gene with a mean FPKM of at least 1 in at least one tissue was considered expressed, and its breadth of expression was recorded as the number of tissues reaching this threshold. A heatmap was drawn with TBtools after row-wise standardisation of the FPKM values (Chen et al., 2020).

### 2.10 Re-analysis of public stress transcriptomes

Responses to abiotic stress were examined in three public peanut RNA-seq datasets. The drought dataset (PRJNA706902) (Zhao et al., 2021) comprises leaves of the drought-tolerant line L422 after 5, 7 and 9 d of water withholding at the pod-setting stage, each with a well-watered control sampled at the same time. The cold dataset (PRJNA751249) (Wang et al., 2021) comprises leaves of two-week-old seedlings of the cold-tolerant cultivar Silihong and the cold-sensitive cultivar Zhonghua 12 after 0, 3, 24 and 48 h at 10 °C. The salt dataset (PRJNA1092346) (Wang et al., 2024) comprises shoots and roots of the salt-tolerant cultivars Yuhua 18 and Huayu 9510 and the salt-sensitive cultivars Puhua 76 and Fenhua 8 after 7 d in nutrient solution containing 1% NaCl, together with untreated controls; the cultivar codes of the runs were matched to cultivar names through the BioSample records. Every condition had three biological replicates, 90 runs in total.

The runs were downloaded from the NCBI Sequence Read Archive, checked with vdb-validate and converted to FASTQ with fasterq-dump (sra-tools v3.4.1); all libraries were paired-end. Reads were quantified against the 101,155 annotated NDH108 coding sequences with Salmon v1.10.3 (Patro et al., 2017), with automatic library-type detection, selective alignment and sequence- and GC-bias correction. All libraries were inferred to be unstranded, and 48% to 68% of the reads were mapped, as expected for an index that lacks untranslated regions. Transcript estimates were summarised to genes with tximport v1.30.0 (Soneson et al., 2015), genes with at least ten reads in fewer than three samples were removed, and differential expression was tested with DESeq2 v1.42.0 (Love et al., 2014) in R v4.3.3. Each treatment was compared with its own control, giving 17 contrasts: drought against the control at each time point, each cold time point against 0 h within each cultivar, and salt against the control within each cultivar, with shoots and roots modelled separately. A GST was considered expressed in a contrast if its mean TPM was at least 1 in either group, and responsive if it was also significant at an adjusted P below 0.05 with an absolute log2 fold change of at least 1; these criteria and the contrasts were fixed before the results were examined. The proportions of responsive genes in core and dispensable OGGs were compared with Fisher’s exact test over combinations of gene and contrast; because the combinations of one gene are not independent, the test is descriptive.

## 3 Results

### 3.1 Identification of *GST*s across the *Arachis* panel

We assembled a panel of 20 *Arachis* genomes covering three genome types, comprising 16 AABB allotetraploids, 3 AA diploids and 1 BB diploid. The tetraploids included several representative cultivars of *A. hypogaea* as well as the wild allotetraploid *A. monticola*, whereas the diploids corresponded to the ancestral lineages of the A and B subgenomes. The species tree inferred from the concatenated whole-proteome alignment (**Figure 1**) placed the three AA diploids as successive basal branches and the BB diploid *A. ipaensis* within the tetraploid clade, with SH-like local supports of 0.90 to 1.00. Because every tetraploid contributes genes of both subgenomes to the concatenated matrix, this tree reflects overall proteome similarity rather than the phylogeny of the A and B lineages, and it was used here to order the genomes in the figures. After identifying *GST* members in each genome, a total of 1,436 GST genes were obtained across the 20 genomes (**Figure 2**, **Supplementary Table S2**). The number of *GST* genes varied considerably among genomes. The tetraploid genomes contained between 53 and 87 genes, with a mean of approximately 79.5, whereas the diploid genomes contained fewer genes, ranging from 36 to 45 in the AA type and 38 in the BB type. The GST number of each tetraploid genome was approximately equal to the sum of one A-subgenome and one B-subgenome diploid, which is consistent with the origin of peanut as an allotetraploid formed through hybridisation between the A- and B-genome diploid ancestors followed by chromosome doubling (Bertioli et al., 2016, 2019).

**Figure 2:**
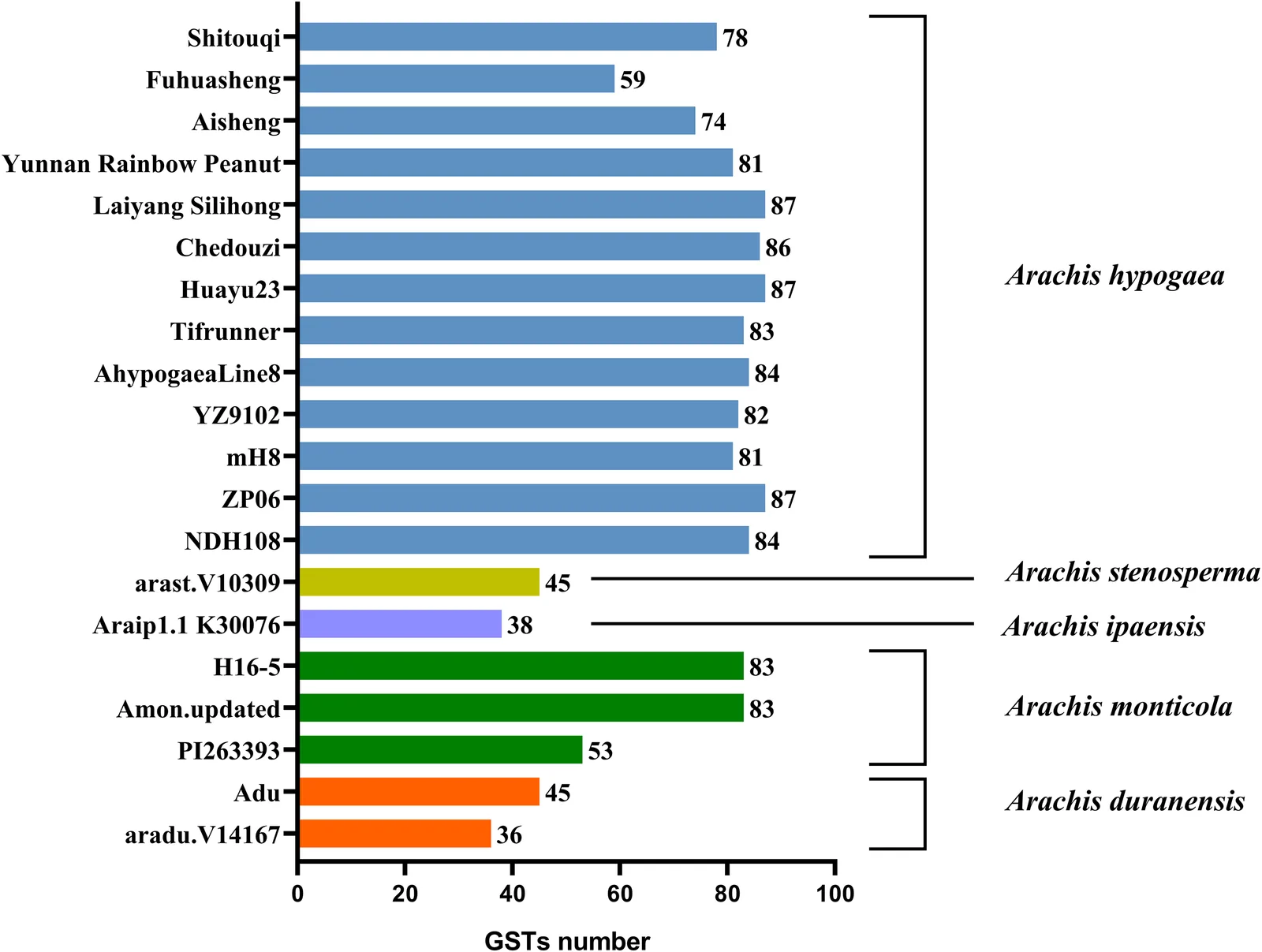
Number of *GST*s identified in each genome. In this horizontal bar chart, the value on each bar is the *GST* count of that genome, and colors denote species.

### 3.2 Gene structure of the peanut *GST*s

The structure of all 1,436 *GST*s was extracted from the GFF3 annotations of the 20 genomes (**Figure 3**, **Supplementary Tables S3**, **S4**). The family was structurally uniform. Median gene length was 1,558 bp (interquartile range 1,168-2,184 bp), median protein length 224 amino acids (221-230) and median total intron length 642 bp (398-1,139 bp), while 85.9% of the genes carried a single intron and 88.0% carried two CDS features. Because the median gene comprised only two CDS features separated by one intron, most of the variation in gene length was contributed by intron length rather than by coding sequence, and the two measures varied in parallel across genomes. Protein length was the most constrained metric, 93.1% of the genes falling between 215 and 239 amino acids and the family as a whole spanning only 182 to 348 amino acids, consistent with the conserved two-domain architecture of the plant *GST*s. Intron number and CDS number were invariant across genomes, the median being one intron and two CDS features in all 20 (**Supplementary Table S4**). Gene length differed modestly among genomes, the median ranging from 1,269 to 1,968 bp, and the four genomes with the shortest medians were the same four whose annotations gave anomalous proximal counts in the duplication analysis described below, so this difference is more likely to reflect annotation than biology.

**Figure 3:**
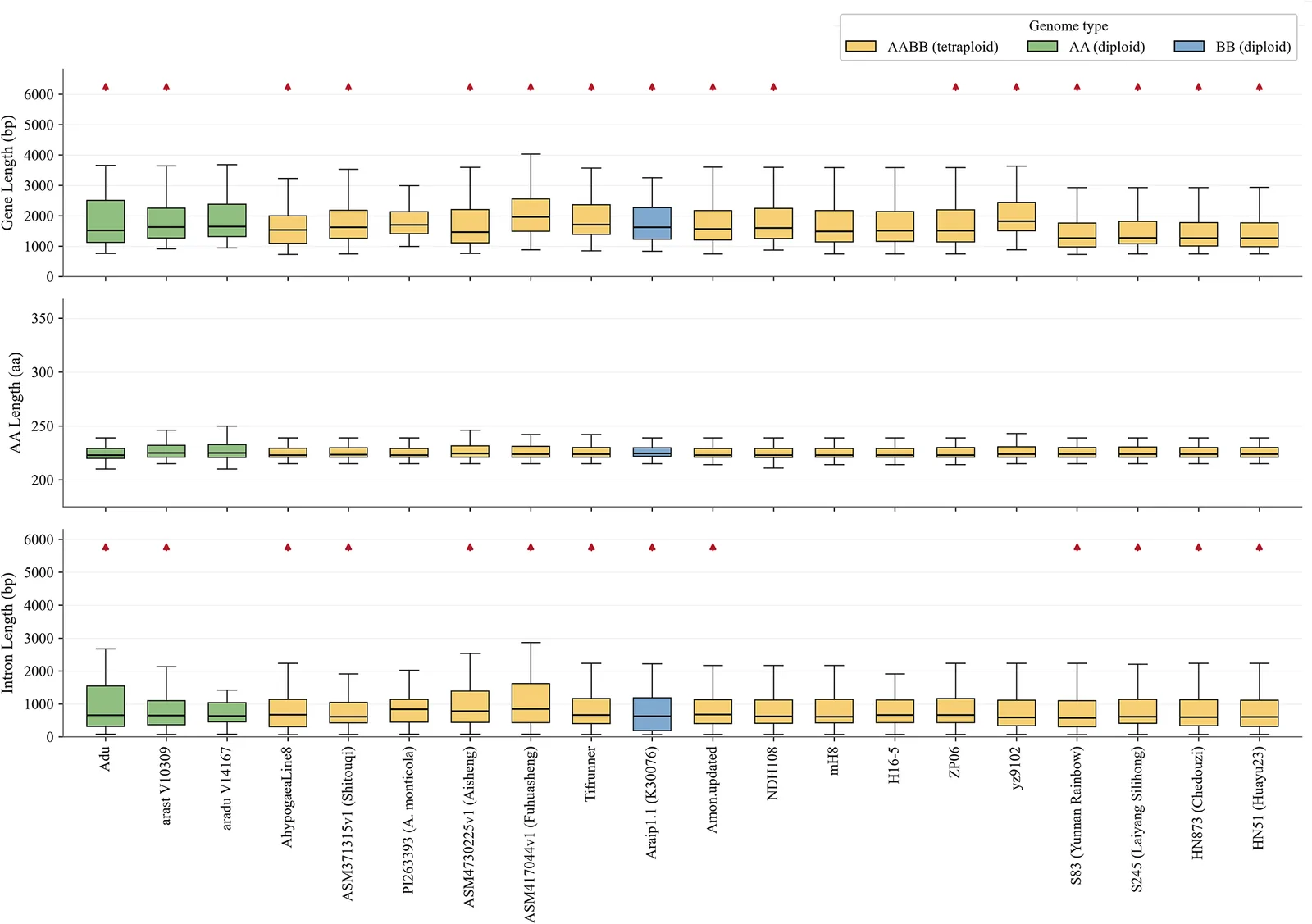
Gene structure of the peanut *GST* family across the 20 genomes. Boxplots give the distribution of gene length, protein length and total intron length among the *GST*s of each genome, with genomes arranged in species-tree order and boxes colored by genome type. Gene length and intron length are clipped at the 98th percentile of the family distribution, 6,500 bp and 6,000 bp respectively, and a red arrow marks a genome containing a gene above the axis. Protein length is plotted from 175 amino acids, the family spanning only 182 to 348 amino acids. Intron number and CDS number are invariant across genomes and are given in **Supplementary Table S4**.

### 3.3 Core and dispensable *GST*s

To examine the conservation of the GSTs across the panel, the 1,436 *GST*s identified in the 20 genomes were assigned to orthologous gene groups, yielding 57 OGGs that constitute the *Arachis* GST pan-gene family (**Supplementary Table S5**). On the basis of the presence of each OGG across the 20 genomes, the 57 OGGs were classified into 18 core OGGs (present in all 20 genomes) and 39 dispensable OGGs (absent in at least one genome), the latter comprising 20 softcore OGGs (present in ≥90% of the genomes, that is 18 to 19 genomes), 10 shell OGGs (present in >10% of the genomes, that is 3 to 17 genomes) and 9 line-specific OGGs (present in ≤10% of the genomes, that is no more than 2 genomes)(**Figure 4A**). The number of OGGs (57) was smaller than the GST count of most tetraploid genomes (53 to 87), indicating that many OGGs contain multiple copies within a genome. At the gene level, the proportion of genes in each category was not proportional to the number of OGGs (**Figure 4B**). Core genes accounted for 44.08% of all GSTs and softcore genes for 42.97%, so that together they represented about 87% of the genes and formed the bulk of the family. Shell genes accounted for 12.12% (174 genes) and line-specific genes for 0.84% (12 genes). The composition of individual genomes illustrated the conservation of the core OGGs across ploidy levels (**Figure 4C**; **Supplementary Table S6**). In the three AA diploids and the single BB diploid, core genes numbered 18 to 19 and the total *GST* complement ranged from 36 to 45, whereas in the AABB tetraploids core genes numbered 29 to 38 and the total complement ranged from 53 to 87. Each diploid carried a single copy of nearly every core OGG, whereas in the tetraploids 225 of the 288 combinations of core OGG and genome carried two copies, consistent with the origin of allotetraploid peanut through the merger of two diploid subgenomes. Each genome carried 0 to 2 line-specific genes. Most GSTs therefore belong to core and softcore OGGs, and variation in OGG content lies mainly in the shell OGGs. Absence of a softcore OGG was often confined to the diploids or to the two tetraploids with the smallest GST complements, Fuhuasheng and *A. monticola* PI263393 (59 and 53 GSTs), eight of the 20 softcore OGGs being absent only from one or two diploids and five only from one or both of these tetraploids; the bearing of this on the categories is considered in Section 4. Structural uniformity declined along the conservation gradient. The proportion of genes with a single intron fell from 90.7% among core genes through 84.0% among softcore genes to 78.2% among shell genes, and the proportion with two CDS features fell correspondingly from 93.7% through 84.8% to 81.6% (**Supplementary Figure S1**; **Supplementary Table S7**). The 12 line-specific genes were too few to establish a trend. Three OGGs departed from the narrow protein-length range of the family, OG0070909 and OG0055613 at approximately 300 amino acids and OG0052282 above 320; each comprises only one or two genes and all three are shell or line-specific.

**Figure 4:**
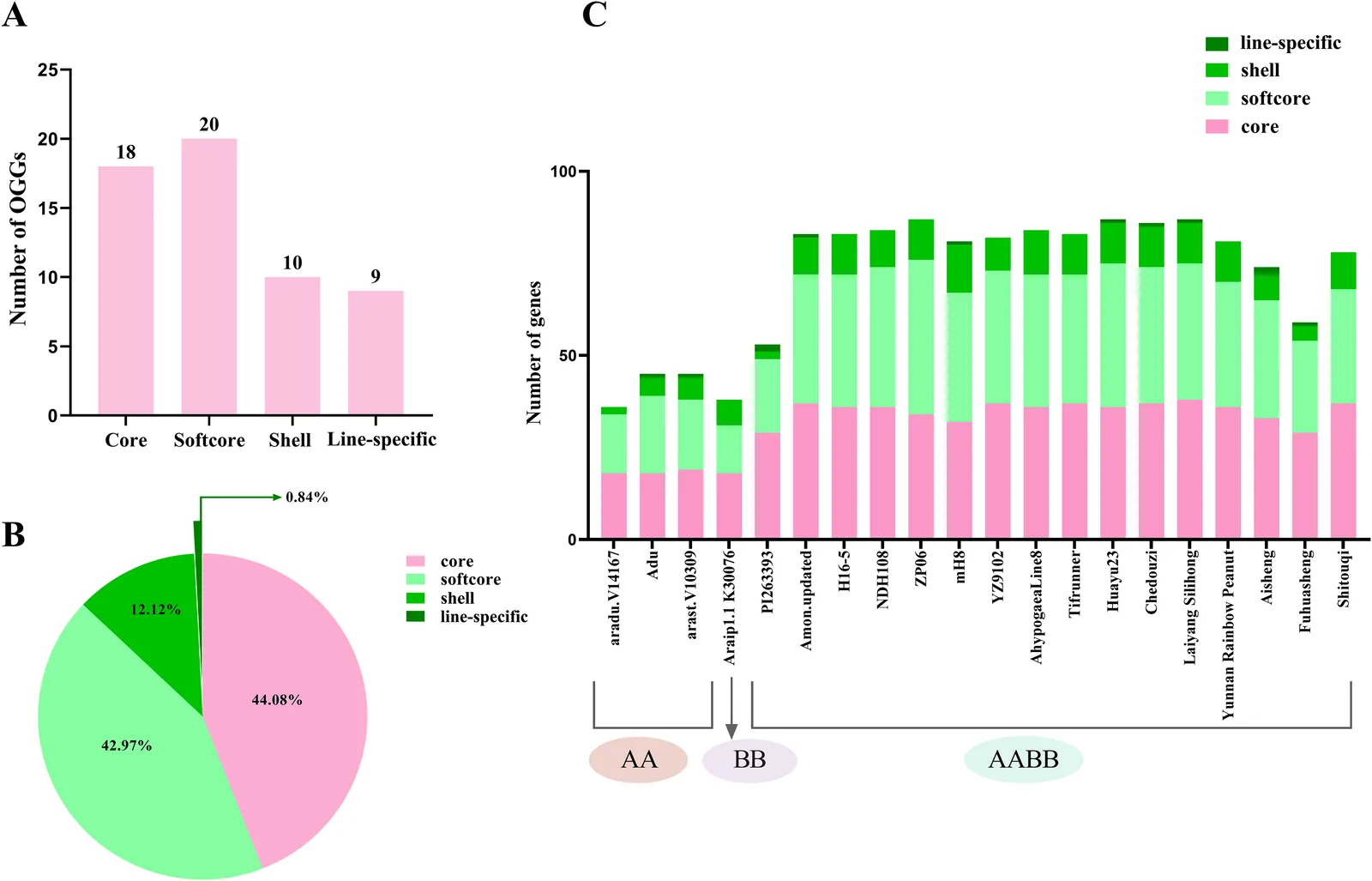
Pan-genome composition of the *GST* family. **(A)** Number of OGGs in the core, softcore, shell and line-specific categories (18, 20, 10 and 9, respectively). **(B)** Pie chart of the proportion of the four categories over all 1,436 *GST* genes (core 44.08%, softcore 42.97%, shell 12.12% and line-specific 0.84%). **(C)** Stacked bar chart decomposing the *GST* count of each genome into the four categories, with the AA, BB and AABB groups indicated below.

### 3.4 Phylogenetic classification and subfamily assignment

To determine the subfamily assignment of the peanut GST OGGs, the representative sequences of the 57 OGGs, listed in **Supplementary Table S5**, were aligned together with 53 *Arabidopsis* GST proteins and used to construct a phylogenetic tree (**Figure 5**). The 57 peanut GST OGGs were distributed among four subfamilies, with 53 in Tau, 2 in Phi, and 1 each in Theta and Lambda. The DHAR, Zeta and TCHQD subfamilies contained no peanut OGGs, and their sectors were defined only by the *Arabidopsis* sequences. This absence follows from the identification criteria, which these classes do not meet, rather than from their absence from the peanut genomes (Section 4). The best BLASTP hit of each of the 1,436 GSTs among the *Arabidopsis* GSTs belonged to the subfamily of its OGG in every case. The Tau subfamily contained 53 of the 57 OGGs, about 93%, and was the largest subfamily, whereas the other subfamilies contained few peanut OGGs. This pattern is consistent with the general situation in plants, in which Tau is the largest *GST* subfamily. Because the Tau subfamily contained the great majority of the peanut GST OGGs, all nine line-specific and nine of the ten shell OGGs belonged to Tau, as did 17 of the 18 core and 18 of the 20 softcore OGGs. Of the other four, the Lambda OGG was core, the Theta OGG and one Phi OGG softcore and the second Phi OGG shell. The peanut *GST* family is therefore dominated numerically by the Tau subfamily, and within this two-domain set the variation in OGG content also lies mainly within Tau.

**Figure 5:**
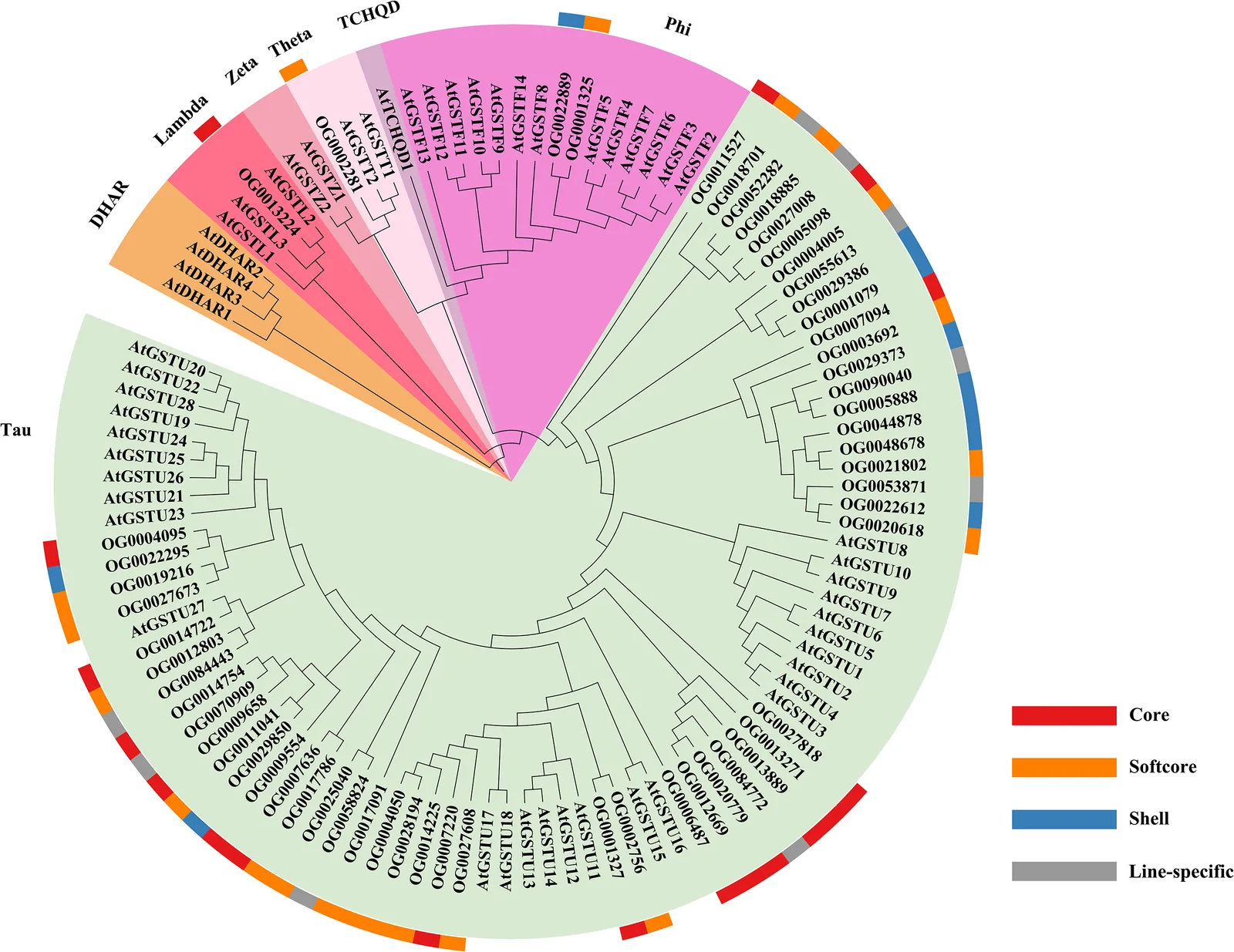
Phylogenetic tree of representative *GST* sequences. Maximum-likelihood tree (IQ-TREE, Q.YEAST+R4, 1,000 ultrafast bootstrap replicates) of one representative sequence per OGG together with the 53 *Arabidopsis* GSTs, background colors distinguish the Tau, Phi, Theta, Zeta, Lambda, DHAR and TCHQD subfamilies, and the outer ring marks the core, softcore, shell and line-specific categories. No peanut OGG falls in the Zeta, DHAR or TCHQD sectors because these classes are not captured by the two-domain identification criteria. Each peanut tip is the representative sequence of one OGG, labelled with the OGG identifier; the representative genes are listed in **Supplementary Table S5**.

### 3.5 Analysis of copy-number variation and protein motifs

To examine the variation in copy number of the peanut GST OGGs among genomes, the copy numbers of the 57 OGGs across the 20 genomes were tabulated (**Figure 6**; **Supplementary Table S8**). Among all 1,140 combinations of OGG and genome, a copy number of 2 accounted for 37.3%, a copy number of 1 for 34.5%, and a copy number of 0 for 23.2%, while copy numbers of 3 and 4 accounted for 3.1% and 1.9%, respectively. In terms of the highest copy number reached by each OGG across genomes, 18 OGGs reached at most 1, 31 reached at most 2, 6 reached at most 3, and 2 reached at most 4. Eight OGGs reached a copy number of 3 or 4, most of them core or softcore OGGs; among them, OG0001327 (core) and OG0001325 (softcore) reached a copy number of 4. The copy number was highest in the core OGGs and decreased towards the dispensable OGGs. In the genomes in which a given OGG was present, the mean copy number was 1.76 for core, 1.66 for softcore, 1.33 for shell and 1.00 for line-specific OGGs. About 67% of the combinations of core OGG and genome had a copy number of at least 2, whereas all line-specific genes were single-copy. When ploidy was taken into account, the mean copy number of core OGGs was approximately 1.0 in the AA and BB diploids and approximately 1.9 in the AABB tetraploids, indicating that the copy number of 2 commonly observed in the tetraploids corresponds to the retention of one copy in each of the A and B subgenomes. Conserved motifs of the representative sequences of the 57 OGGs were analysed with MEME (**Figure 7**). Most of the representative sequences, which belong mainly to the Tau subfamily, contained a similar set of motifs with a conserved composition and arrangement, whereas the few sequences in the Lambda and Phi subfamilies differed in motif composition from those of the Tau subfamily. The motif composition was consistent among phylogenetically close sequences, in agreement with the subfamily classification described above.

**Figure 6:**
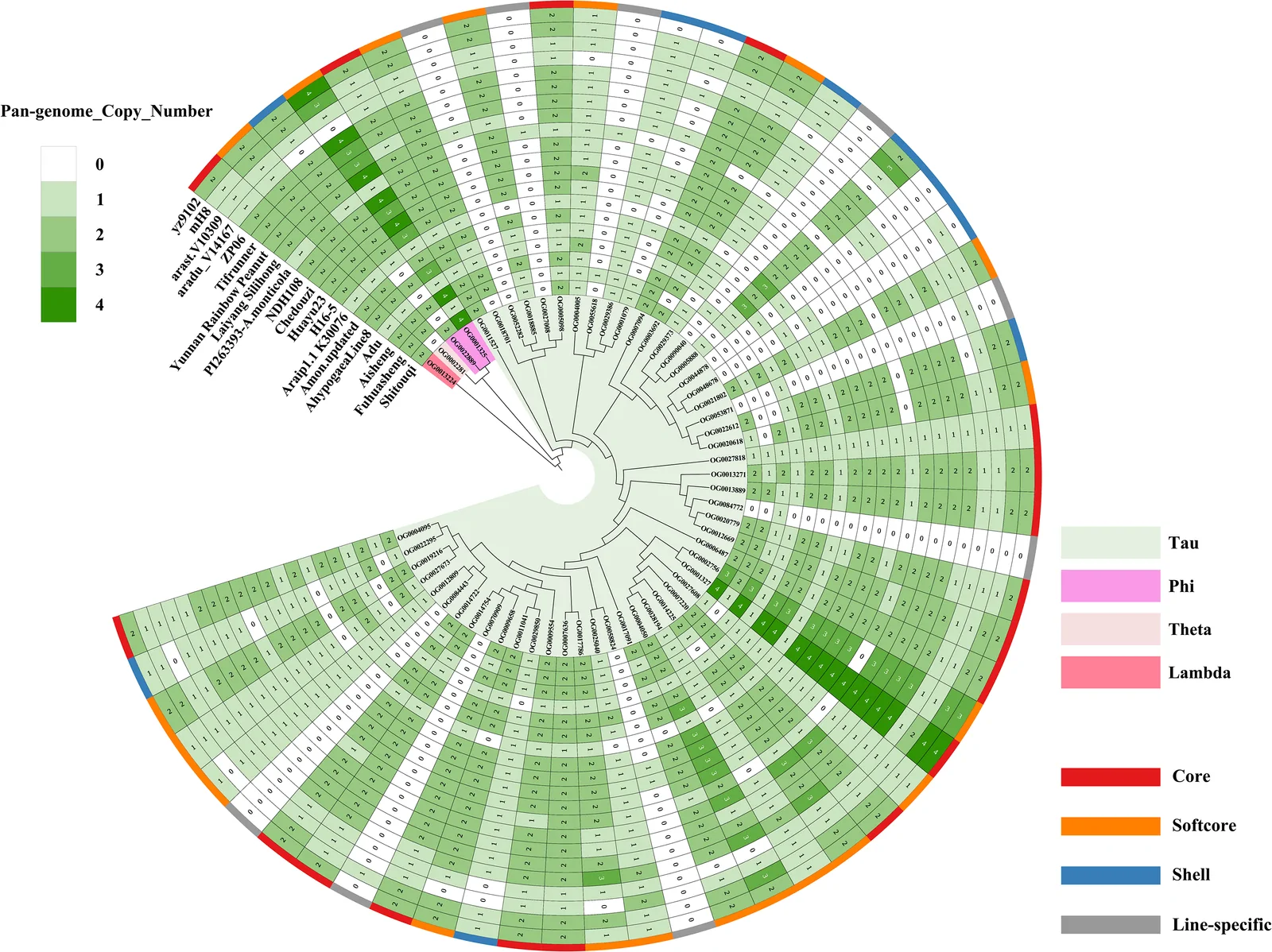
Distribution of *GST* copy number across the 20 genomes. The heatmap ring outside the circular tree of the representative sequences, whose tips are labelled with OGG identifiers, shows the copy number of each OGG in every genome (0-4), background colors distinguish subfamilies, and the outermost ring marks the conservation category.

**Figure 7:**
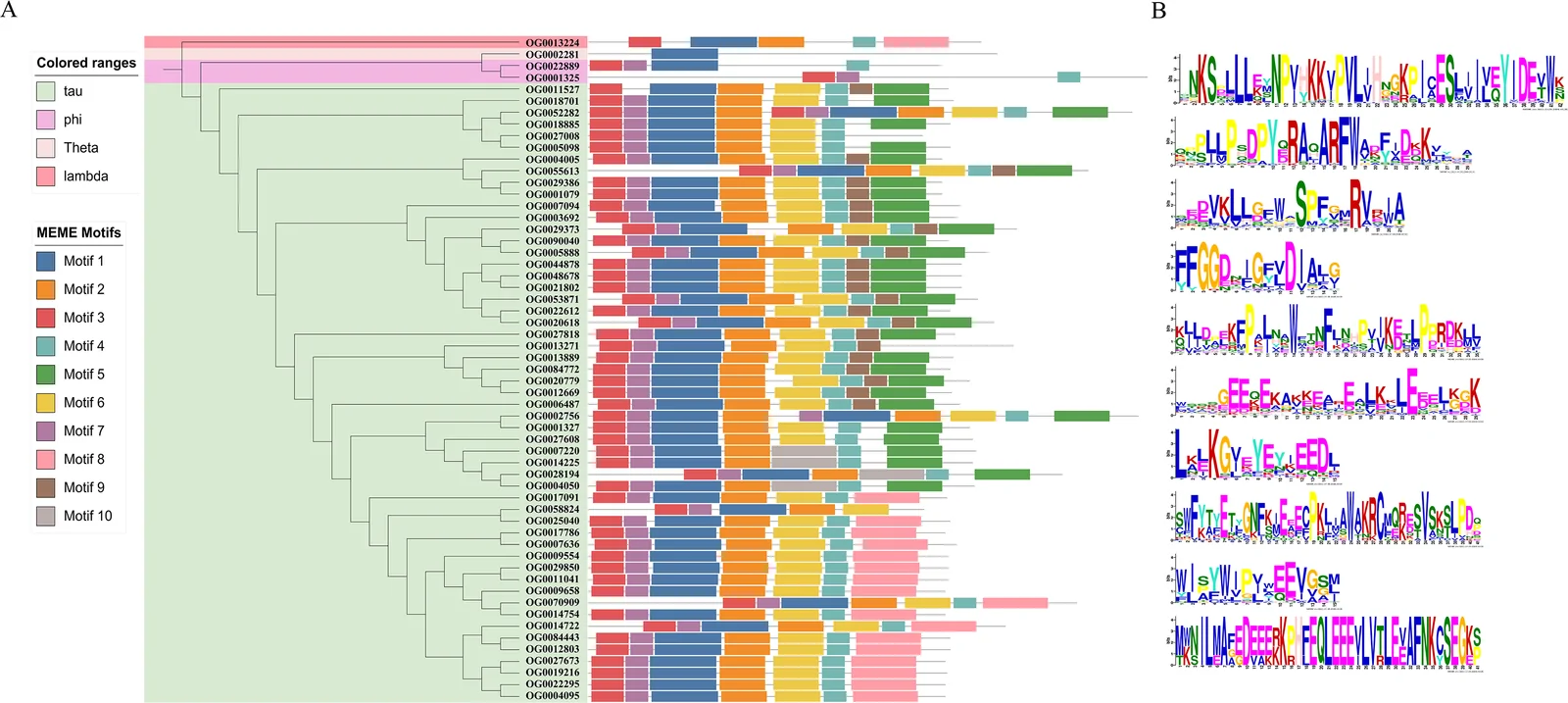
Conserved motif analysis of GSTs. **(A)** Tree of the representative sequences of the 57 OGGs, labelled with OGG identifiers, and the distribution of the ten MEME-identified motifs along each sequence, with background colors distinguishing subfamilies. **(B)** Sequence logos of the ten motifs.

### 3.6 Gene duplication and expansion

To resolve the duplication modes underlying the expansion of the peanut *GST* family, we classified the duplication origin of all 1,436 *GST* genes with MCScanX, which distinguished five modes, namely WGD/segmental, tandem, proximal, dispersed and singleton (**Figure 8A**). At the whole-family level, WGD/segmental duplication accounted for the largest fraction, comprising 782 genes (54.46%), followed by tandem duplication with 287 genes (19.99%). Dispersed and proximal duplications contributed 187 genes (13.02%) and 173 genes (12.05%), respectively, and singletons numbered only 7 (0.49%). This composition indicates that the present size of the peanut *GST* family was shaped mainly by WGD/segmental and tandem duplication, which together accounted for 74.45% of the genes. When the genes were partitioned by pan-genome category, the mode composition varied across categories (**Figure 8B**; **Supplementary Table S9**). Among the 633 core genes, WGD/segmental duplication comprised 413 genes, accounting for 65.2% of these genes and clearly exceeding tandem (80), dispersed (78) and proximal (58) duplications, with only 4 singletons. Among the 617 softcore genes, WGD/segmental duplication comprised 324 genes (52.5%) and remained the principal source, while tandem duplication rose to 133 genes (21.6%). Among the 174 shell genes, tandem duplication comprised 73 genes (42.0%), exceeding WGD/segmental duplication (41 genes, 23.6%), and proximal and dispersed duplications numbered 33 and 26, respectively. The 12 line-specific genes were too few to support a separate trend. WGD/segmental duplicates thus predominated among core and softcore genes, whereas tandem duplicates outnumbered them among shell genes. Resolving the same classification by genome (**Figure 8C**; **Supplementary Table S10**) separated the diploids from the tetraploids. In the four diploid genomes whole-genome/segmental duplication accounted for 4.4% to 15.8% of that genome’s *GST*s, that is 2 to 6 genes, whereas in the 16 tetraploid genomes it accounted for 35.8% to 67.5%, that is 19 to 58 genes; tandem duplication ran in the opposite direction, from 36.8% to 55.6% in the diploids down to 5.8% to 25.6% in the tetraploids. In a tetraploid the A and B subgenomes supply each other with collinear partners, so most homoeologous GST copies are classified as WGD/segmental, whereas in a diploid only older duplications can form collinear blocks. Because the classifier assigns each gene to a single mode with WGD/segmental taking precedence, a tandem duplicate that also has a homoeologous partner is counted as WGD/segmental, which lowers the tandem share of the tetraploids. The contrast between the two ploidy levels therefore reflects mainly the homoeologous pairs created by allopolyploidisation; the classification describes present gene positions and does not by itself establish when the tandem and segmental duplications arose. Resolved by OGG (**Figure 9**; **Supplementary Table S11**), the same gradient held for individual OGGs, the median within-OGG WGD/segmental share being 71% for core, 52% for softcore and 17% for shell OGGs and the median tandem share 9%, 17% and 45%; the category-level percentages given above pool the genes of all OGGs in a category. The largest OGGs were dominated by whole-genome/segmental duplication, OG0001327 comprising 56 such genes of 66, whereas several shell OGGs were tandem-driven, among them OG0029373 and OG0022889. Proximal duplicates were absent from three genomes and numbered one in a fourth, while these same four genomes carried the highest dispersed counts of the panel; because the proximal mode depends on gene ordering within the annotation, this pattern is more consistent with differences between the annotations than with a biological difference, and the family-level proximal share of 12.05% should be read with that in mind.

**Figure 8:**
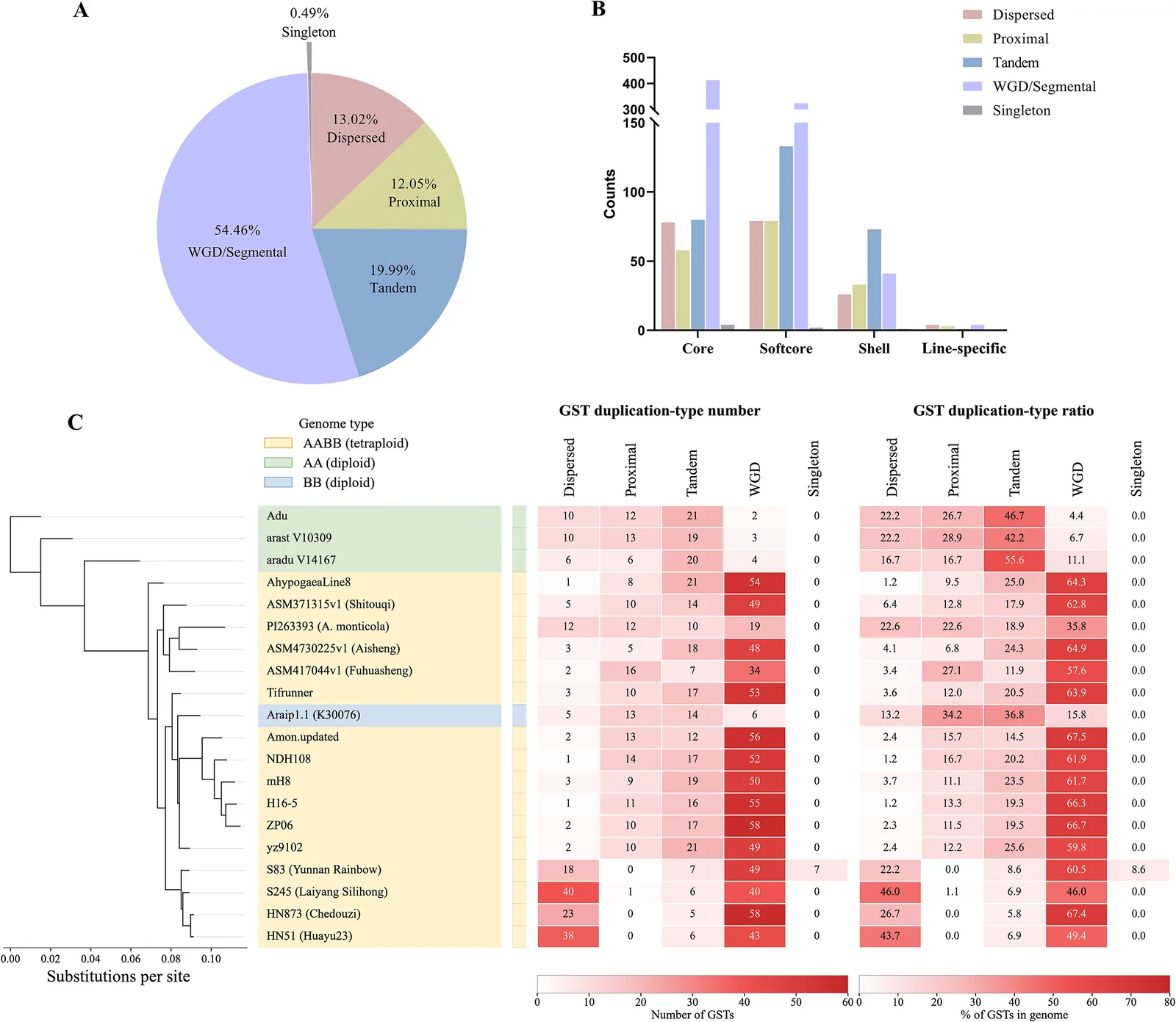
Gene-duplication modes of the *GST*s. **(A)** Pie chart of the overall proportions of the five modes (WGD/segmental 54.46%, tandem 19.99%, dispersed 13.02%, proximal 12.05% and singleton 0.49%). **(B)** Counts of the dispersed, proximal, tandem, WGD/segmental and singleton modes among the genes of the core, softcore, shell and line-specific categories. **(C)** The classification resolved by genome. Genomes are ordered by the OrthoFinder species tree, with branch lengths in substitutions per site, and the band to the left of the matrices marks the genome type. The left matrix gives the number of *GST*s assigned to each mode in each genome and the right matrix the corresponding percentage of that genome’s *GST*s. WGD denotes WGD/segmental duplication.

**Figure 9:**
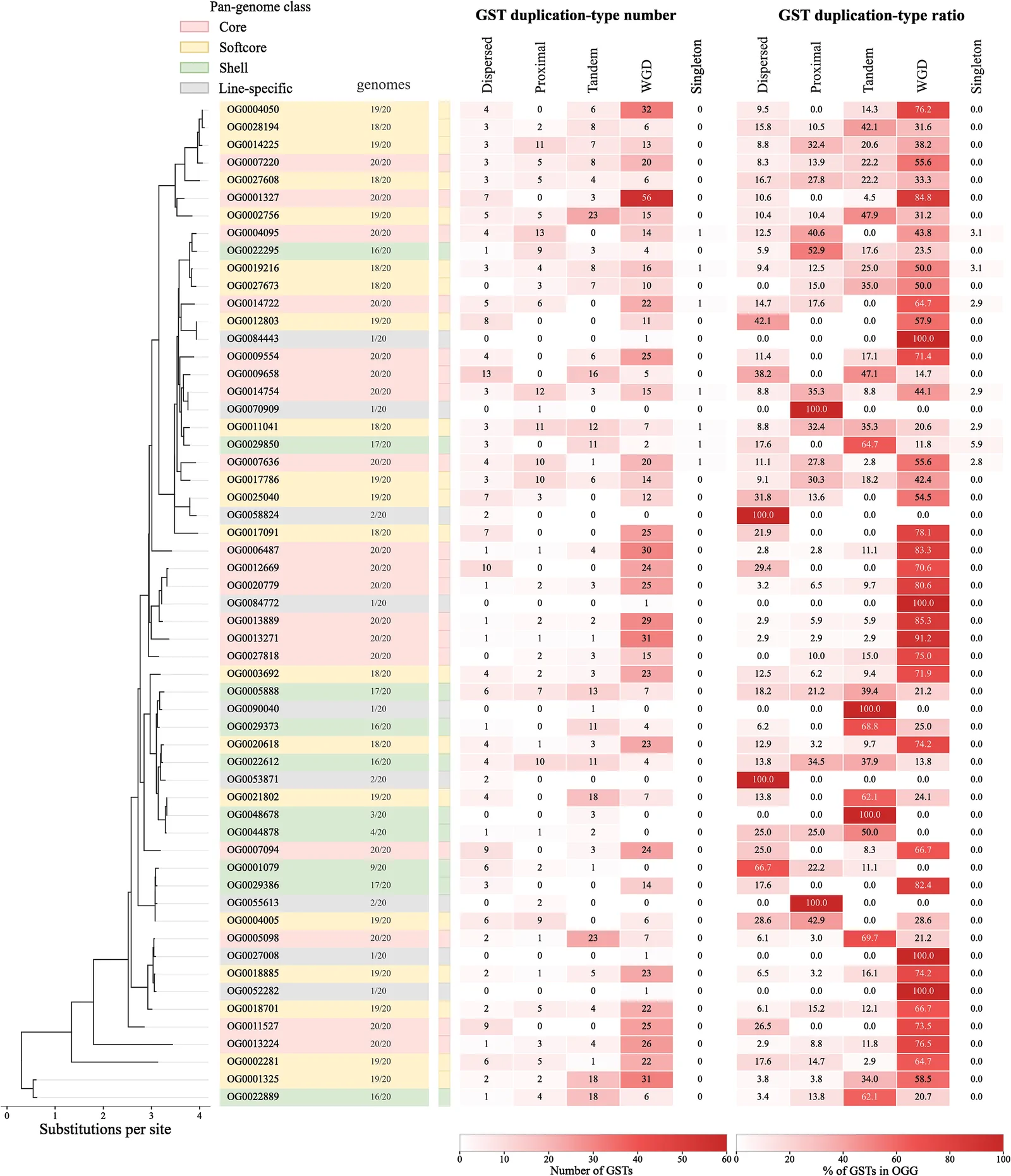
Gene-duplication modes of the 57 *GST* OGGs. OGGs are ordered by the backbone tree of the representative sequences, the band to the left of the matrices marks the pan-genome class and the adjacent column gives the number of genomes the OGG occurs in. The left matrix gives the number of *GST*s assigned to each of the five duplication modes within the OGG and the right matrix the corresponding within-OGG percentage. WGD denotes WGD/segmental duplication.

### 3.7 Collinearity of the peanut *GST*s

Collinear *GST* pairs were first sought within each genome (**Figure 10**; **Supplementary Table S12**). The 20 genomes together yielded 430 such pairs, and their number differed sharply with ploidy, from 7 to 32 per tetraploid genome but only 1 to 3 per diploid. Because the tetraploids carry two subgenomes that diverged before their merger, most of their within-genome collinear GST pairs connect homoeologous positions in the A and B subgenomes, 356 of the 423 pairs in the tetraploids, a source of collinearity that the diploids lack. Collinearity was then examined between genomes, comparing the two diploid progenitors *A. duranensis* and *A. ipaensis* with the wild allotetraploid *A. monticola* and the cultivated tetraploid Tifrunner (**Figure 11**; **Supplementary Figure S2**; **Supplementary Table S13**). Of the 237 collinear *GST* pairs whose members belonged to the same OGG, 125 linked chromosomes of the same subgenome and 112 linked the A and B subgenomes. Core and softcore OGGs accounted for 119 of the 125 same-subgenome pairs and shell OGGs for the remaining six, so the core and softcore OGGs occupy syntenic positions shared by the diploid progenitors and the tetraploids. The wild and the cultivated tetraploid behaved almost identically against either progenitor. Compared with *A. duranensis*, both *A. monticola* and Tifrunner returned 37 pairs with the same class composition, 21 core, 14 softcore and 2 shell, and compared with *A. ipaensis* both returned 33 pairs, 19 core, 13 softcore and 1 shell in *A. monticola* against 20, 12 and 1 in Tifrunner. The OGGs involved were nevertheless not entirely the same. Within the A subgenome OG0006487 and OG0007220 were collinear with *A. duranensis* only in *A. monticola* and OG0012803 only in Tifrunner, and within the B subgenome four OGGs were collinear with *A. ipaensis* only in *A. monticola* and two only in Tifrunner. Synteny of the family is therefore broadly conserved between the wild and cultivated tetraploids while differing at individual loci. The same relationships within the Tifrunner genome alone are shown as a circular plot (**Supplementary Figure S3**), in which most links span different chromosomes, consistent with the predominance of WGD/segmental duplication at the family level.

**Figure 10:**
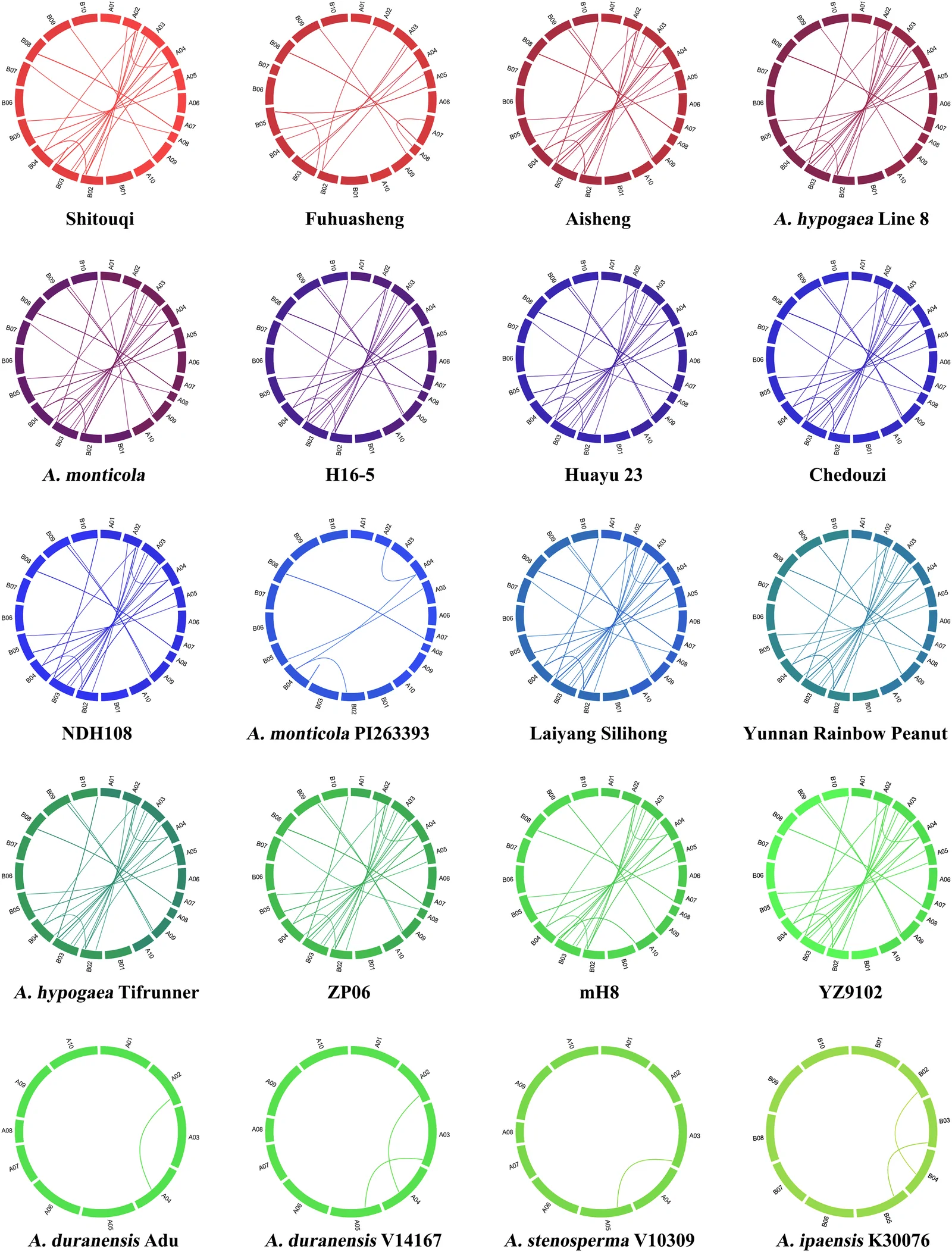
Collinear *GST* pairs within each of the 20 *Arachis* genomes. In each circular plot the ring shows the chromosomes of that genome, labelled A01-A10 and B01-B10 by subgenome, and links connect the collinear gene pairs whose two members are both *GST*s. The 16 allotetraploid genomes are arranged in the first four rows and the four diploid genomes in the last row, and each genome is drawn in its own color.

**Figure 11:**
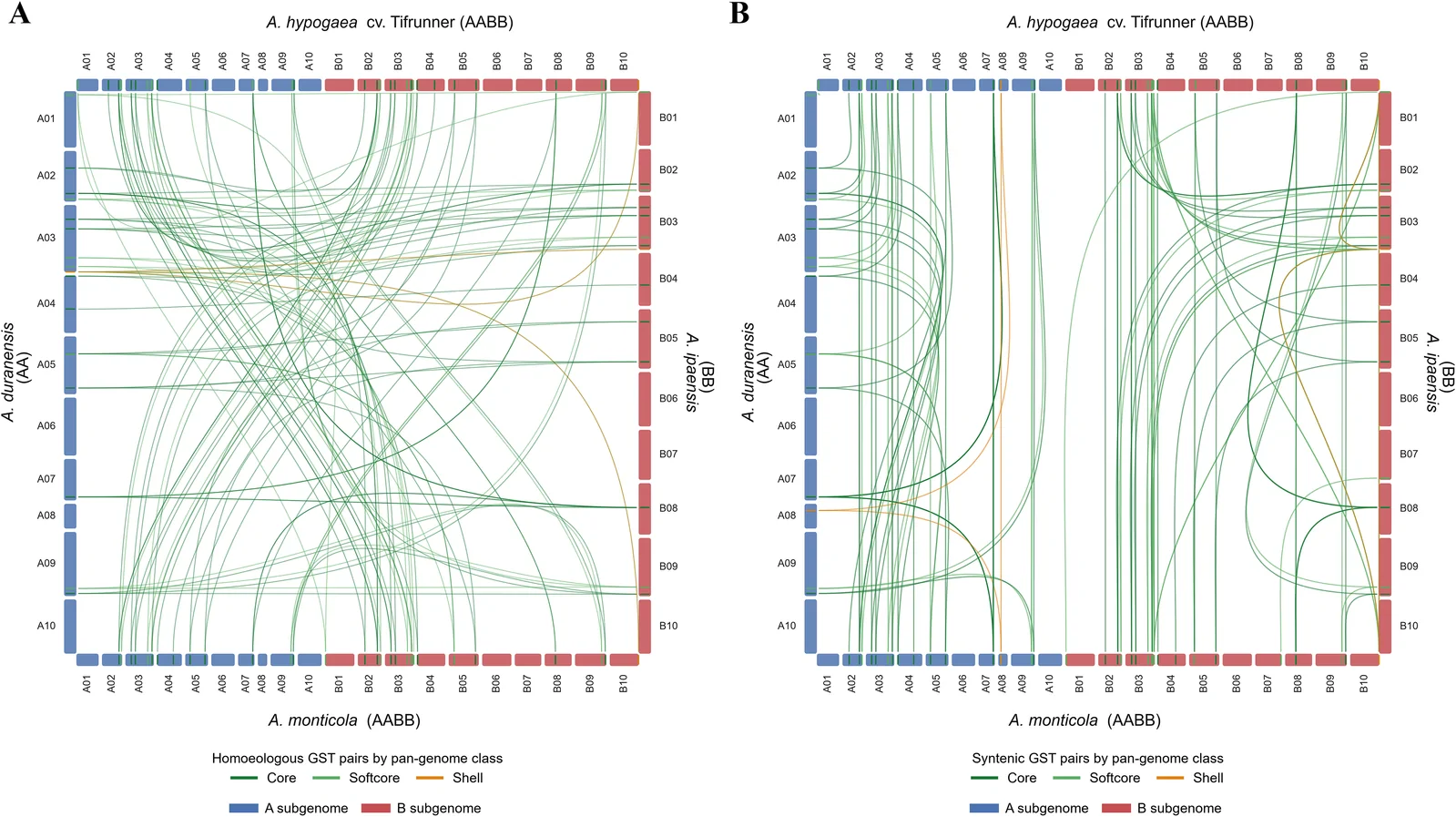
Collinearity of the *GST*s between the diploid progenitors and the tetraploids. *A. duranensis* (AA) and *A. ipaensis* (BB) are placed on the left and right axes and *A. hypogaea* cv. Tifrunner (AABB) and *A. monticola* (AABB) on the top and bottom axes, chromosome bars are colored by subgenome, and the ticks within each bar mark the positions of the *GST*s. Lines connect collinear *GST* pairs whose two members belong to the same OGG and are colored by the pan-genome class of that OGG. **(A)** Pairs linking the A and B subgenomes, which are homoeologous rather than orthologous. **(B)** Pairs linking chromosomes of the same subgenome.

### 3.8 Analysis of natural selection on the peanut *GST*s

To assess the selective pressure acting on the peanut *GST* family during its evolution, all pairwise comparisons among the genes within each OGG were performed on the basis of the orthology results of the 20 genomes, giving 23,399 within-OGG gene pairs, and the non-synonymous substitution rate (Ka), the synonymous substitution rate (Ks) and their ratio (Ka/Ks) were calculated for each pair. Ka, Ks and Ka/Ks were each averaged over the pairs within the OGG. Of the 57 OGGs, six contained a single gene and therefore yielded no pair, and in a further five every pair returned an undefined ratio, either because the two sequences were identical or because Ks was zero; the remaining 46 OGGs were retained for the analysis of Ka/Ks. The mean Ka/Ks value was below 1 in 43 of these 46 OGGs, consistent with purifying selection predominating at the OGG level (**Figure 12A**; **Supplementary Table S14**). The same held for individual pairs (**Supplementary Table S15**). Of the 14,626 pairs with a finite ratio, 13,697 (93.6%) had Ka/Ks below 1, and the 7,509 pairs that departed significantly from neutrality by Fisher’s exact test (P < 0.05) all had Ka/Ks below 1, whereas none of the 929 pairs with a ratio above 1 or of the 619 pairs with Ks = 0 did so (smallest P 0.29 and 0.36, respectively). Three OGGs gave mean values slightly above 1, namely OG0013224 (1.12), OG0018701 (1.07) and OG0017091 (1.04). In each of the three the elevated ratio was consistent across the OGG rather than driven by a single outlying pair, but no individual pair in any of them departed significantly from neutrality by Fisher’s exact test, the smallest P value being 0.22, and each pair carried only five to ten substitutions; these point estimates therefore do not constitute evidence of positive selection. The distribution of the pairwise values within each OGG (**Figure 13**) shows the basis of these means. Ka and Ks were concentrated near zero in most OGGs, consistent with the 34.9% of pairs whose coding sequences were identical, whereas the OGGs with the largest membership, among them OG0001327 and OG0002756, spanned a wider range of Ks and account for most of the divergence observed within the family. Core and dispensable OGGs were then compared (**Figure 12B**). Neither Ka, Ks nor Ka/Ks differed significantly between the two classes, with P values of 0.451, 0.715 and 0.854 from Welch’s t-test and 0.657, 0.760 and 0.991 from the Mann–Whitney U test, so no difference in selective constraint between core and dispensable OGGs was detected.

**Figure 12:**
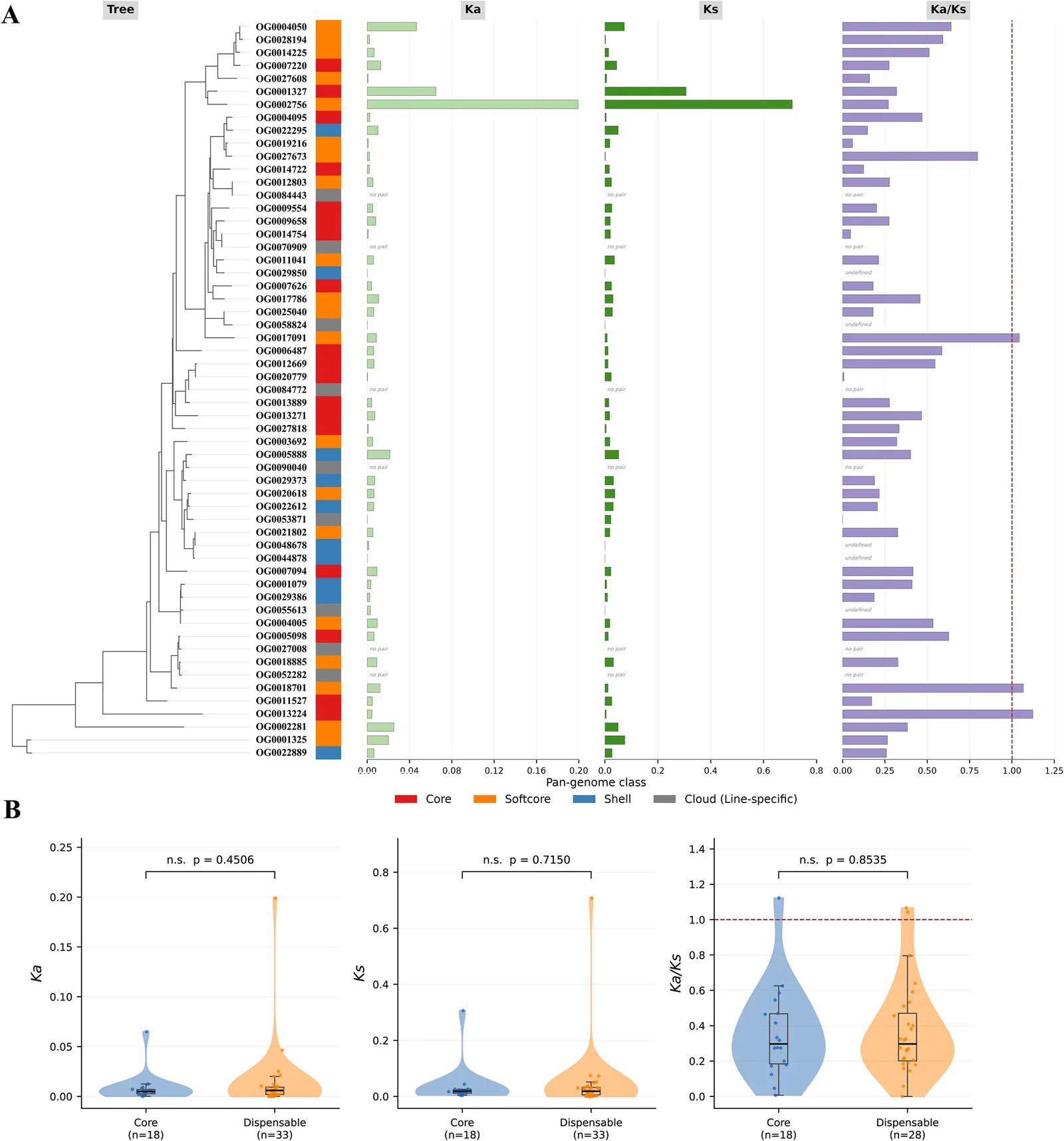
Selection pressure on the *GST* OGGs. **(A)** Backbone tree of the representative sequences with the mean Ka, Ks and Ka/Ks of each OGG, the color strip marking the pan-genome class. Six single-gene OGGs yield no pair and are marked no pair, and five OGGs in which every pair returned an undefined ratio are marked undefined; the dashed line marks Ka/Ks = 1 and the three panels have different x-axis ranges. **(B)** Violin plots comparing the core and dispensable groups for Ka, Ks and Ka/Ks, one point per OGG, with P values from Welch’s t-test (Ka 0.451, Ks 0.715, Ka/Ks 0.854) and the Mann–Whitney U test (0.657, 0.760 and 0.991); none is significant. The Ka and Ks panels include the 51 OGGs containing at least one pair and the Ka/Ks panel the 46 for which the ratio could be estimated.

**Figure 13:**
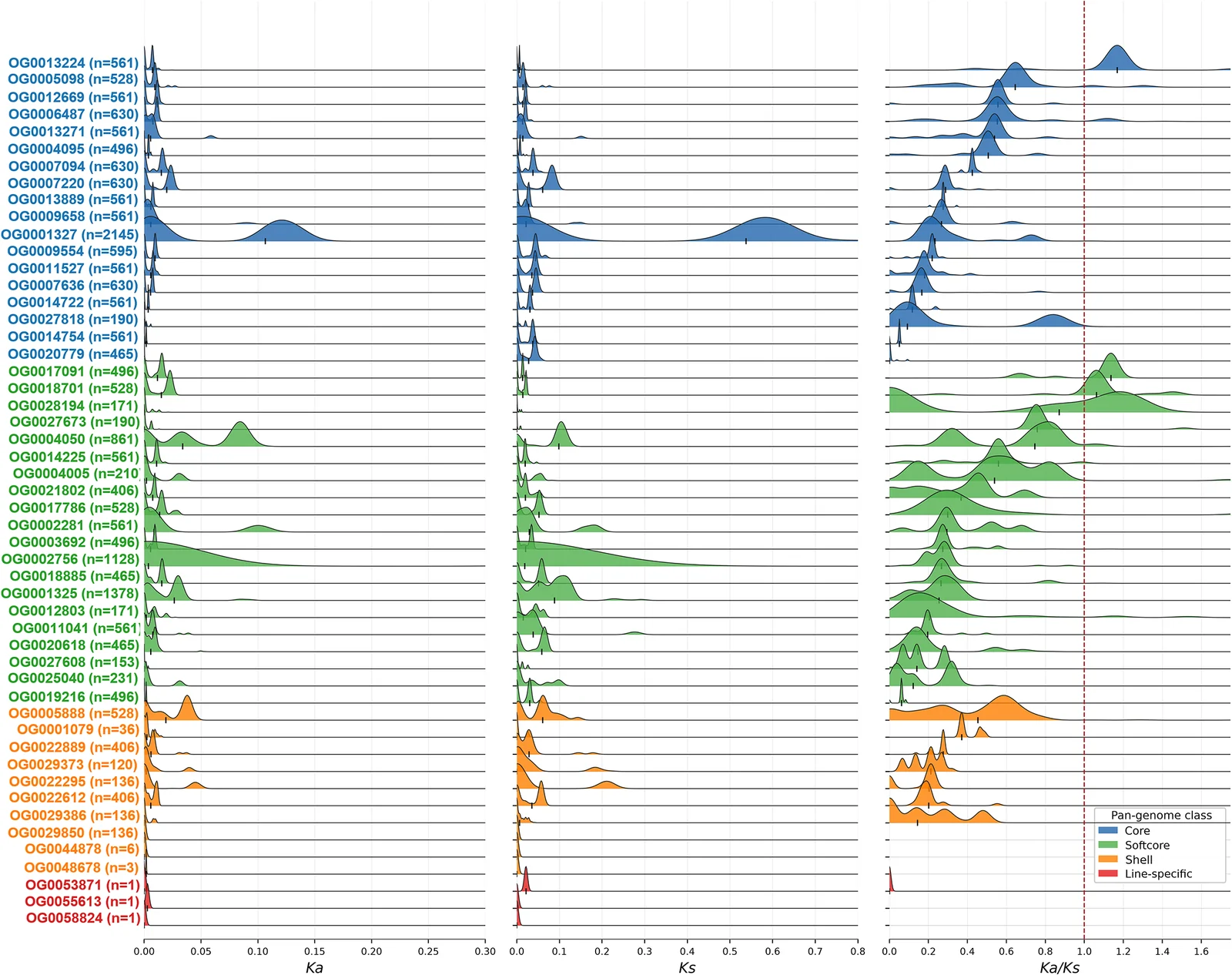
Distribution of Ka, Ks and Ka/Ks within each *GST* OGG. Ridgeline plots give the density of the pairwise values over the gene pairs of each OGG for the 51 OGGs containing at least one pair, with the number of pairs in parentheses and the rows colored by pan-genome class. The tick on each baseline marks the median of that OGG and the dashed line marks Ka/Ks = 1. Identical pairs are included in the Ka and Ks panels but excluded from the Ka/Ks panel, where the ratio is undefined, as are the 619 pairs with Ks = 0 and Ka > 0.

### 3.9 Tissue expression profiles

Of the 84 GSTs of NDH108, the 83 with expression records belong to 18 core, 20 softcore and 7 shell OGGs; NDH108 carries no line-specific GST (**Figure 14**; **Supplementary Table S16**). Seventy-four of the 83 reached an FPKM of at least 1 in at least one tissue. All 35 core genes were expressed, whereas the nine silent genes comprised eight softcore genes, among them both copies of OG0017091 and of OG0017786, and the single NDH108 gene of the shell OGG OG0029386. Breadth of expression followed the same order, 14 of the 35 core genes reaching an FPKM of 1 in at least 18 of the 22 tissues against 8 of the 38 softcore and 3 of the 10 shell genes, the last belonging to OG0005888 and OG0022889. The calyx was the most frequent tissue of peak expression, for 22 of the 74 expressed genes, followed by the leaf and the branch root with ten each. The highest values were reached in the calyx by NDH07G23720.1 and NDH18G19620.1 of OG0019216 (980 and 417 FPKM) and NDH04G19220.1 of OG0020779 (473 FPKM), in the 30-day seed by NDH14G01830.1 and NDH04G01310.1 of OG0001327 (523 and 253 FPKM) and in the pistil by NDH03G24050.1 of OG0012669 (310 FPKM). Copies of the same OGG did not necessarily share a profile. Of the 28 OGGs with two or more expressed copies in NDH108, 15 had copies peaking in different tissues, and in OG0004050 one copy reached 88 FPKM in the petal while the other two were silent. Whether the calyx-preferential members act in floral organs cannot be decided from these data.

**Figure 14:**
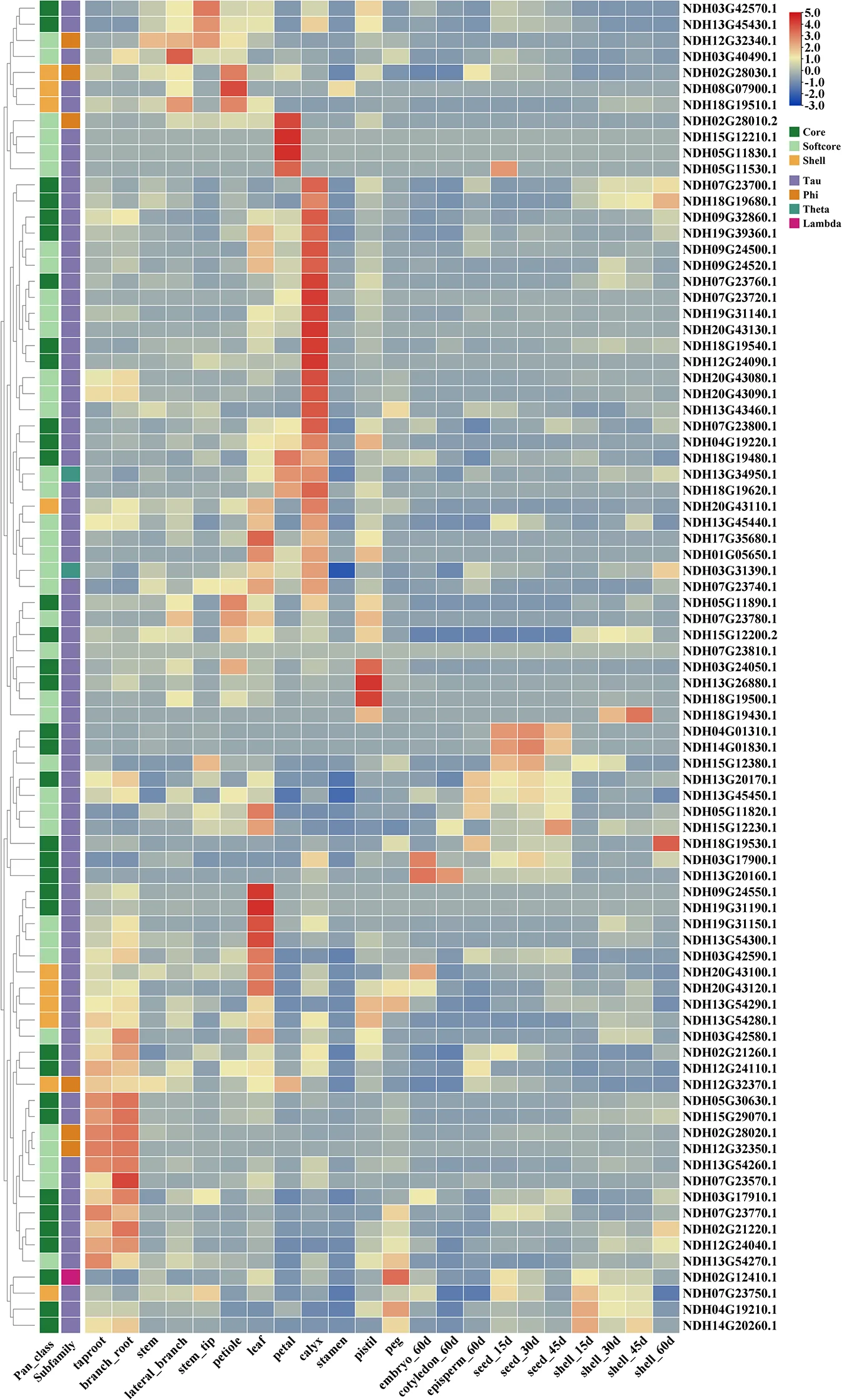
Expression of the NDH108 GSTs across 22 tissues. Rows are the 83 NDH108 GSTs with expression records, labelled with their gene identifiers, and columns are the 22 tissues; the strips on the left give the pan-genome category and subfamily of each gene, whose OGG is listed in **Supplementary Table S16**. Mean FPKM values of three replicates were standardised by row, and rows were hierarchically clustered.

### 3.10 Responses of the GSTs to drought, cold and salt stress

The 84 NDH108 GSTs were examined in 17 treatment-control contrasts from three public RNA-seq datasets (**Figure 15**; **Supplementary Tables S17**, **S18**). Seventy-six genes were expressed in at least one dataset and 74 responded in at least one contrast, 49 to drought, 61 to cold and 60 to salt, while 37 responded to all three, 17 of them core, 12 softcore and 8 shell genes. Of the 485 significant combinations of gene and contrast, 381 (78.6%) were up-regulations. Six of the nine genes that were silent in the tissue atlas were not expressed in any stress sample either.

**Figure 15:**
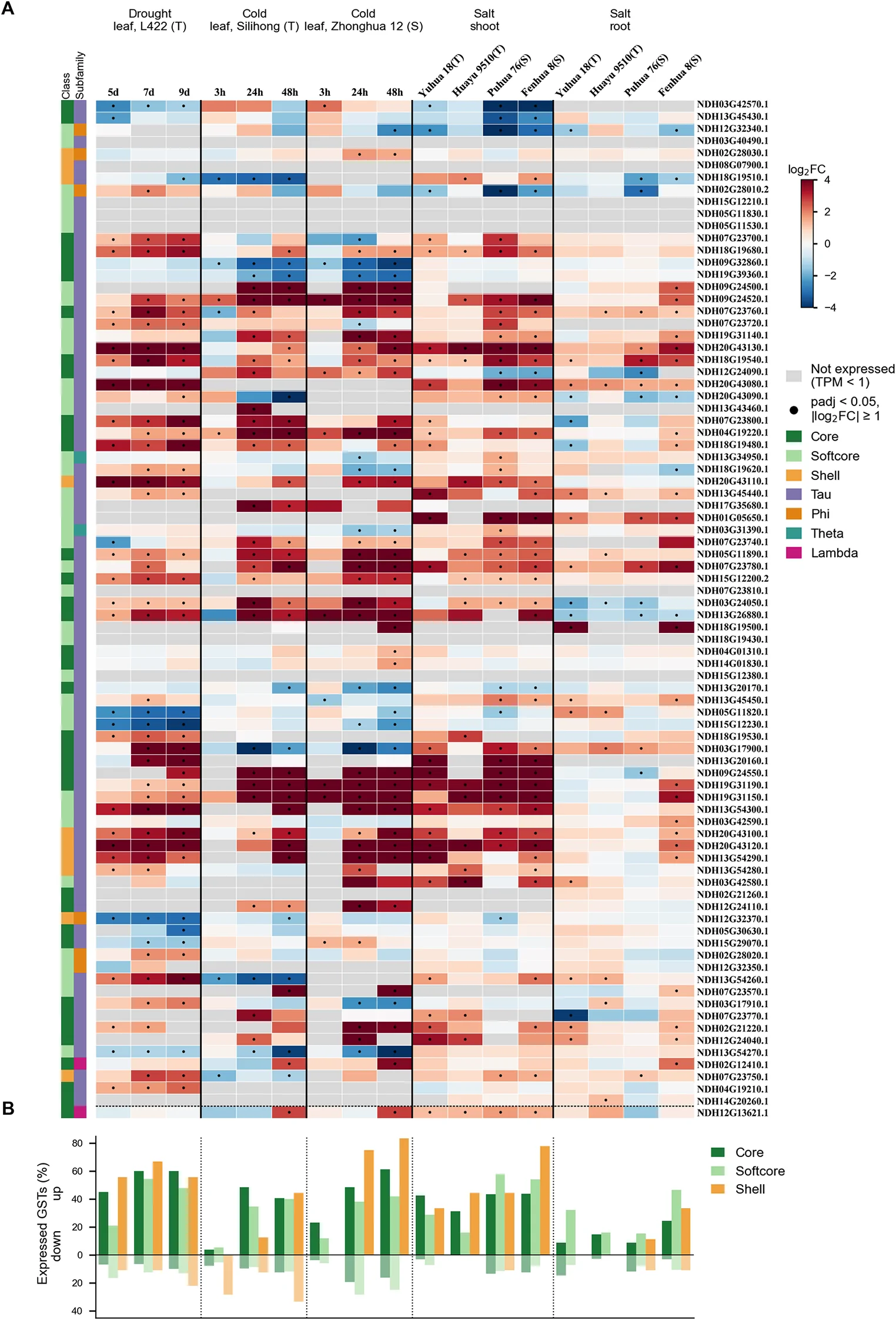
Responses of the NDH108 GSTs to drought, cold and salt stress in three public RNA-seq datasets. **(A)** Log2 fold change of each treatment relative to its control. Rows follow the order of **Figure 14**, with NDH12G13621.1, which has no atlas record, added below the dashed line; columns are grouped by dataset, and (T) and (S) mark tolerant and sensitive genotypes. Grey cells denote genes not expressed in the contrast (mean TPM below 1 in both groups), dots mark an adjusted P below 0.05 with an absolute log2 fold change of at least 1, and colours are capped at ±4. The strips on the left give the pan-genome category and subfamily. **(B)** Percentage of the expressed GSTs of each category that were up-regulated (above zero) or down-regulated (below zero) in each contrast.

The extent of the response depended on the duration of stress and on the tissue. Under cold, 13.5% of the expressed GSTs had responded at 3 h in Silihong and 21.7% in Zhonghua 12, against 48.4% to 56.1% and 68.3% to 73.8% at 24 and 48 h. Under drought the share rose from 48.4% at 5 d to 68.3% and 67.7% at 7 and 9 d. Under salt, 27.3% to 61.5% of the expressed GSTs responded in shoots and 14.7% to 41.4% in roots. The sensitive genotypes responded more broadly in the cold dataset at every time point and in salt-treated shoots, where 61.5% and 61.2% of the expressed GSTs responded in Puhua 76 and Fenhua 8 against 27.3% and 40.0% in Huayu 9510 and Yuhua 18, whereas roots showed no consistent difference. With one or two genotypes per tolerance class, these differences are descriptive.

The share of responsive genes did not differ between core and dispensable OGGs in any dataset (**Figure 15B**), being 62.9% and 60.2% of the expressed combinations of gene and contrast under drought (Fisher’s exact test, P = 0.76), 50.8% and 47.1% under cold (P = 0.52) and 34.4% and 38.7% under salt (P = 0.33). Copies of the same OGG differed mainly in whether they responded. Of the 408 combinations of OGG and contrast with at least two expressed copies, 102 combined responsive and unresponsive copies, and in only two, OG0004095 in Zhonghua 12 at 24 h and OG0020618 at 9 d of drought, did copies change in opposite directions.

## 4 Discussion

All results of this study are computational, derived from published assemblies, annotations, expression atlases and RNA-seq data, and none was validated experimentally. Previous identifications of the *GST* family in peanut and most plants have relied on a single reference genome (Wang et al., 2019b). Here, 1,436 GSTs were identified across 20 *Arachis* genomes and assigned to 57 OGGs, of which some were conserved across all genomes (core) and the rest varied in presence and absence among genomes (dispensable). Because of this presence/absence variation, any single genome lacks part of the dispensable OGGs and cannot represent the complete membership of the family. Using OGGs as the unit of analysis, members were divided by conservation level into core, softcore, shell and line-specific categories, and the duplication origin, selective pressure and tissue expression were then examined separately for each category, comparisons that a single reference genome does not support (Tong et al., 2025; Wang et al., 2026).

The identification criteria define the scope of these conclusions. Requiring both PF02798 and PF00043 excluded GST classes whose C-terminal domain is poorly matched by PF00043. Ten of the 53 *Arabidopsis* GSTs, among them both Zeta and both Theta members, AtDHAR3 and AtGSTL2, gave no PF00043 hit, and BLASTP with the *Arabidopsis* GSTs as queries recovered 29 Tifrunner proteins that were absent from the 1,436 genes although they shared 51% to 77% identity with an *Arabidopsis* GST, five of them Zeta, five DHAR, two TCHQD, three Theta, six Lambda and eight Phi (**Supplementary Table S19**). Most of these carried no PF00043 hit, while the two TCHQD and eight Phi proteins hit both models but lacked specific CD-Search hits to both domains. The absence of DHAR, Zeta and TCHQD OGGs therefore reflects the criteria rather than the genomes, and the 1,436 genes represent the canonical two-domain GSTs, chiefly Tau, rather than the complete family. This criterion accounts at least in part for the difference from the 163 genes in six classes reported in a single-reference survey of peanut (Wang et al., 2019b). The subfamily composition, Tau predominance and pan-genome categories reported here apply to this two-domain set, and whether the omitted classes show the same distribution across genomes remains untested.

The pan-genome categories need to be read with the composition of the panel in mind. Four of the 20 genomes are diploids of the progenitor lineages and two tetraploids represent the wild *A. monticola*, so the categories describe conservation across the genus panel rather than across accessions of cultivated peanut; eight of the 20 softcore OGGs were absent only from one or two diploids, and when the classification was repeated for the 16 tetraploids alone with the same proportional thresholds, 27 OGGs were core (**Supplementary Table S5**). The categories are also properties of OGGs and not of gene copies. A core OGG may carry one copy in one genome and four in another, and copies of the same OGG can differ in expression, so the conservation of an OGG does not imply that each of its copies is conserved. Absence is further affected by assembly and annotation and by the identification criteria. Five softcore OGGs were absent only from Fuhuasheng or *A. monticola* PI263393, the two tetraploids with the fewest GSTs, and 23 OGGs contained 212 members that failed the GST criteria in the genomes concerned; scoring these as present would move nine OGGs into a more conserved category, five of them from softcore to core. The core-softcore boundary and part of the shell are therefore sensitive to data quality, and the categories are best treated as graded conservation within this panel.

The 1,436 *GST*s were structurally uniform across the 20 genomes, the median member carrying a single intron, two CDS features and a protein of 224 amino acids, and protein length varying least of the metrics examined. Structural uniformity nevertheless declined from the core through the softcore to the shell OGGs, in both intron and CDS number. The shell OGGs are also those in which tandem duplicates predominate, so structural divergence and small-scale duplication coincide in the variable part of the family. Whether this reflects relaxed constraint on younger copies or less reliable annotation of them cannot be distinguished with the present data.

WGD/segmental duplicates made up 54.46% of the GSTs and tandem duplicates 19.99%. Cultivated peanut is an allotetraploid formed by the recent merger of its two diploid ancestors, *A. duranensis* and *A. ipaensis* (Bertioli et al., 2016), and the homoeologous copies created by this recent polyploidization, together with collinear blocks retained from earlier legume polyploidization events, are the likely source of most WGD/segmental assignments (Zhuang et al., 2019). The mode composition differed among conservation categories. Core and softcore were dominated by whole-genome/segmental duplication (65.2% within the core), whereas shell was dominated by tandem duplication (73 tandem versus 41 whole-genome/segmental). One interpretation is that genes retained as homoeologous pairs in collinear blocks persist across genomes and so fall into the core, whereas tandem copies are gained and lost more readily and so vary in presence. The classification records present gene positions rather than the timing of duplication, and in the tetraploids it assigns tandem copies with a homoeologous partner to WGD/segmental, so this interpretation is not tested directly by the data. The association between duplication mode and conservation agrees with the observation in the barley bHLH and tea WRKY pan-genomes that whole-genome duplication shapes a conserved core while small-scale duplication contributes variable members (Tong et al., 2025; Wang et al., 2026). In the allotetraploid quinoa, segmental duplication was likewise reported as the main source of GST expansion, with Tau and Phi the largest classes (Tiwari et al., 2023). Nine of the ten shell OGGs belong to Tau, so in this two-domain set the tandem-rich variable component lies almost entirely within that subfamily.

Collinearity analysis placed the duplication results in a positional context. Within the tetraploid genomes 7 to 32 collinear *GST* pairs were recovered against only 1 to 3 in the diploids, and between genomes about half of the collinear *GST* pairs linked the A and B subgenomes. Both observations follow from the recent merger of the two progenitor genomes, which created homoeologous copies of much of the family, and they are consistent with the predominance of whole-genome/segmental duplication in the tetraploids and its near absence from the diploids, where tandem duplication instead accounts for the largest share. The syntenic positions occupied by the core and softcore OGGs were shared by the progenitors and by both the wild and the cultivated tetraploid, while individual OGGs differed between *A. monticola* and Tifrunner, so the positions of the conserved OGGs are stable across the panel and variation is concentrated at particular loci.

The mean Ka/Ks value was below 1 in 43 of the 46 OGGs for which the ratio could be estimated, consistent with predominantly purifying selection. The pattern held for individual gene pairs as well as for OGG means. Of the pairs with a finite ratio, 93.6% had Ka/Ks below 1, and every pair departing significantly from neutrality had a ratio below 1, so the OGG means are not an artefact of averaging. Pairwise ratios over whole coding sequences cannot, however, detect selection acting on a few sites or along particular lineages, which would require site- or branch-specific models. Three OGGs gave point estimates slightly above 1, but the underlying pairs carried few substitutions and none departed significantly from neutrality, so these values do not support a claim of positive selection and are better read as the imprecision expected of ratios estimated from a handful of substitutions. When the categories were collapsed into core and dispensable, none of Ka, Ks and Ka/Ks differed between the two groups, so the present data do not resolve a difference in selective constraint between the conserved and the variable components, in contrast to the relaxation reported for dispensable bHLHs in the barley pan-genome (Tong et al., 2025). A likely reason is the composition of the panel, in which 34.9% of the 23,399 within-OGG pairs were identical in coding sequence, because most of the 20 genomes belong to the same cultivated species, so the divergence available for the comparison is limited and the test has little power. Resolving this would require a panel spanning greater phylogenetic distance rather than a reanalysis of these genomes.

In NDH108 every core gene was expressed and broadly expressed genes were more frequent among core than among softcore and shell genes, whereas all silent genes belonged to softcore or shell OGGs. This agrees with observations in barley bHLH and tea WRKY, in which broadly and highly expressed members are mostly core and dispensable members are more weakly and tissue-specifically expressed (Tong et al., 2025; Wang et al., 2026). Two qualifications apply. Copies of the same OGG often differed in their peak tissue, so expression is a property of genes rather than of OGGs. The data also come from one cultivar and cannot show whether the expression of an OGG varies among accessions, which would require transcriptomes of several lines.

Several genes stand out as candidates for experimental study, as hypotheses rather than demonstrated functions. These are the broadly and highly expressed core genes NDH04G19220.1 (OG0020779) and NDH07G23700.1 (OG0004095), which peaked in the calyx; NDH03G24050.1 (OG0012669), which peaked in the pistil; the two seed-expressed copies of OG0001327, the largest OGG of the family; and NDH07G23720.1 (OG0019216), the most highly expressed GST in the atlas. Their involvement in floral development, seed development or redox homeostasis is suggested only by expression and conservation and remains to be tested.

The stress transcriptomes provide an independent view of the same genes. Most NDH108 GSTs responded to at least one of drought, cold and salt, and up-regulation predominated, but core and dispensable GSTs responded at similar rates, so these data neither support nor contradict a link between pan-genome category and stress responsiveness. As in the tissue atlas, copies of the same OGG often differed, here mainly in whether they responded, which again places the response at the level of genes rather than OGGs. NDH20G43130.1 (OG0021802), whose protein is identical to that of AhGST23 and is its reciprocal best BLASTP hit, was up-regulated in 12 of the 17 contrasts, including all drought and salt-treated shoot contrasts; silencing of AhGST23 has been reported to increase the sensitivity of peanut to drought and salt (Li et al., 2026). These comparisons have clear limits. The three datasets differ in genotype, tissue, developmental stage and treatment, each tolerance class is represented by one or two genotypes, and the responses are changes in transcript abundance. They therefore identify candidates for functional testing and do not demonstrate stress functions.

## 5 Conclusion

In this study, a pan-genome analysis of the *GST* family based on orthologous gene groups (OGGs) was conducted computationally across 20 *Arachis* genomes. A total of 1,436 two-domain GSTs were identified and assigned to 57 OGGs, which were classified by conservation level into core, softcore, shell and line-specific categories. The family was structurally uniform, and structural uniformity declined from the core to the shell OGGs. WGD/segmental duplicates were the most frequent mode, predominating among core and softcore genes, whereas tandem duplicates predominated among shell genes, and WGD/segmental duplicates were far more frequent in the tetraploids than in the diploids, reflecting their homoeologous subgenomes. Core and softcore OGGs occupied syntenic positions shared with the diploid progenitors, with about half of the syntenic links connecting the A and B subgenomes. The mean Ka/Ks value was below 1 in 43 of the 46 OGGs for which it could be estimated, consistent with predominantly purifying selection, with no difference detected between core and dispensable OGGs. In the 22-tissue atlas of NDH108 all core genes were expressed and the silent genes belonged to softcore or shell OGGs. In three public stress transcriptomes most NDH108 GSTs responded to drought, cold or salt, at similar rates in core and dispensable OGGs. The 20-genome OGG analysis thus describes variation in GST content, duplication mode and genomic organisation across the *Arachis* panel that a single reference genome does not capture, and the candidate genes it identifies require experimental validation.

## Data Availability

The original contributions presented in the study are included in the article/**Supplementary Material**. Further inquiries can be directed to the corresponding authors.

## Supplementary material

Supplementary Figure 1 Gene structure of the peanut *GST* family across the 57 OGGs, with OGGs arranged in backbone-tree order within three blocks corresponding to the core, the softcore, and the shell together with the line-specific OGGs.

Supplementary Figure 2 Dot plots of the six pairwise comparisons among *A. duranensis*, *A. monticola*, *A. hypogaea* cv. Tifrunner and *A. ipaensis*, with all collinear gene pairs in grey and the *GST* pairs belonging to the same OGG marked and colored by pan-genome class, filled symbols denoting pairs within a subgenome and open symbols pairs between the A and B subgenomes.

Supplementary Figure 3 Collinear GST pairs within the cultivated peanut reference genome Tifrunner, drawn with the Advanced Circos module of TBtools in which the outer ring shows the Tifrunner chromosomes and red links connect the collinear GST gene pairs.
